# The Impact of Visitors on Non-Primate Species in Zoos: A Quantitative Review

**DOI:** 10.3390/ani13071178

**Published:** 2023-03-28

**Authors:** Ellen Williams, Violet Hunton, Geoff Hosey, Samantha J. Ward

**Affiliations:** 1 Department of Animal Health, Behaviour & Welfare, Harper Adams University, Newport TF10 8NB, UK; 2 Deane Campus, University of Bolton, Bolton BL3 5AB, UK; 3 School of Animal, Rural & Environmental Sciences, Brackenhurst Campus, Nottingham Trent University, Southwell NG25 0QF, UK

**Keywords:** visitor effect, non-primate species, zoo animal welfare, zoo visitors

## Abstract

**Simple Summary:**

Visitors in zoos have a variable impact on animals; principally categorised as positive, negative or neutral. This paper quantifies the impact of visitors in non-primate species, based on 105 papers found in the literature. In total, there were 252 non-primate species studied. The number of papers published has increased since 2012, with a range of animal groups now assessed (including avian, reptilian, amphibian, fish, mammalian and invertebrates). Amphibians responded negatively to visitors more frequently than would be expected, birds responded neutrally more frequently than would be expected and fish responded neutrally and ‘unknown’ more frequently than would be expected. A number of animal-based metrics have been used to assess the impacts of visitors on animals, with measures used varying across taxa. It is recommended that moving forwards researchers incorporate a suite of measures, particularly those which are meaningful in terms of being representative of individual animal experiences and animal welfare, collected in a manner which should capture those metrics accurately.

**Abstract:**

Visitors are a prominent feature in the lives of zoo animals, and their presence can cause a range of impacts on zoo animals (typically classed as positive, negative or neutral impacts), commonly referred to as the ‘visitor effect’. This paper quantitatively collates the literature on the visitor effect in non-primate species, investigates the types of measures used to assess impacts of visitors on animals and considers whether impacts vary across non-primate species in zoos. In total, there were 105 papers which had investigated the impact of zoo visitors on 252 non-primate species/species groups. There has been a steady increase in visitor effect research in zoos since 2012 and this body of work incorporates species from avian (28% study species), reptilian (9%), amphibian (2%), fish (4%) and invertebrate taxa (1%). However, there is still a bias towards mammalian species (56%). The response to visitors varied across taxa. Amphibians responded negatively to visitors more frequently than would be expected by chance (*p* < 0.05), birds responded neutrally more frequently than would be expected by chance (*p* < 0.05) and fish responded neutrally and ‘unknown’ more frequently than would be expected by chance (*p* < 0.05). This review highlighted a number of animal-based metrics which have been used to assess the impacts of visitors on animals, with measures used varying across taxa. Moving forwards, it is recommended that moving forwards researchers incorporate a suite of measures, incorporating those which are meaningful in terms of being representative of individual animal experiences and animal welfare, collected in a manner which should capture those metrics accurately.

## 1. Introduction

Visitors are a prominent feature in the lives of zoo animals, with millions of people visiting zoos annually around the world [1]. Zoo visitors are often aspects of a zoo animal’s environment that animals cannot control and are identified as a potential stressor. There is a lot of variation in stimuli from zoo visitors, in terms of their behaviour, the noise they make and the way they interact with animals in zoos. Lack of control is cited as one of the key welfare issues for many animals within zoos [2]. Human–animal interactions (HAIs) within zoos come in a range of forms, both physical (e.g., physical encounters) and non-physical (animals and humans being in close proximity). HAIs within zoos typically include keeper–animal interactions [3], animal ambassador encounters where animals come into close proximity with zoo visitors [4] and general visitor presence within zoos and the associated stimuli [5]. The focus of this paper is on the impact of zoo visitors on animals, and thus it is considered to be, and will be referred to as, the ‘visitor effect’ throughout this work. Visitor effect research in zoos focuses on two main areas: (i) the impact of animal behaviour on zoo visitor experiences and (ii) the impact of zoo visitors on animals [6]. The focus of this paper is on the latter.

There is a large and extensive body of work on the impact of zoo visitors on non-human primates, which began in the 1980s [7] and has accelerated since 2008 when Hosey [8] created a model of human–animal relationships based on knowledge from human–animal relationships in farm animals. More recently, the importance of HAIs has been recognised and the topic advanced, with them being incorporated into the most recent iteration of the five domains model of animal welfare [9]. The initial field of research which coined the visitor effect was predominantly focused on primate species. It highlighted the potential for zoo visitors to be detrimental to zoo primate experiences [10] and suggested zoo primates may be particularly sensitive to the presence of zoo visitors [7]. However, it has been recognised that primates have a unique relationship with zoo visitors; visitors are often drawn to primates in zoos due to their complex behavioural repertoires. Zoo visitors are also drawn to active animals [5,11] and active animals will increase visitor dwell time at enclosures [12]. Studies have reported changes in primate behaviour in response to visitors, with visitors being recognised as a stimulant for some primates [13,14,15] but a negative stressor for others [16,17,18].

Zoo visitors provide three main types of stimuli for animals: visual, auditory and olfactory, and there are considered to be three principal impacts of visitors on zoo animals [19]. Negative impacts are when the visitor acts as a source of stress to the animals, which is usually evidenced by responses such as an increase in visitor avoidance [9], increased stereotypies [20] or increased vigilance [5]. Positive impacts are described as where the visitor is a positive stimulant for the zoo animals, usually evidenced by animals paying increased interest in visitors/visitor areas [21,22], working to gain attention from visitors [23] or moving to be closer to visitors [14,24]. Finally, visitors may have no or neutral impact whereby the animals show no behavioural or physiological changes in response to them [25,26]. However, although this sounds straightforward, researchers have suggested that the visitor effect may be more complex than was first postulated. Animal behaviour may be affected by differing levels of human presence, with some species principally showing increased responses to both low and high numbers of visitors but reduced responses to intermediate levels of visitors [27].

Whilst research across primates and other taxa has shown the adaptability of zoo animals and highlighted their ability to habituate to the presence or absence of zoo visitors [5,28,29,30], it is widely accepted that zoo visitors have varying impacts on zoo animals. The zoo animal–visitor dynamic is complex and there are a number of factors which are likely to impact the valence of these interactions for zoo animals, including but not limited to, previous experience with zoo visitors, behavioural ecology, individual personality and rearing history, enclosure design, husbandry and presence or absence of enrichment [5,10,20,22,31,32].

Different aspects of visitor presence may also have differing effects on animals. Whilst it is likely that the number of visitors will be positively correlated with noise created by visitors this is not always the case [33]. Furthermore, animals may be relatively unaffected by the presence/absence of zoo visitors and the low-level noise they produce through normal conversation but may be more affected by loud or erratic behaviour [34]. The low-level background noise produced by zoo visitors may in fact be beneficial for animals, through the manner in which it can disguise other noise pollution within the zoo environment [35]. However, primates in particular appear to be particularly negatively affected by loud groups of noisy visitors [6]. Similar negative impacts of people on primates have also been observed in ecotourism settings [36]. Understanding whether zoo animals behave differently to different aspects of zoo visitor presence is important, as it enables an opportunity to start to understand what it is about zoo visitors that can make them negative for animals. Understanding negative stimuli enables targeted mitigation efforts, which is important in evidence-based welfare-friendly management of zoo animals.

Our knowledge of the variable impacts of zoo visitors on primate species is extensive; however, the importance of undertaking similar research into the plethora of highly variable non-primate and non-mammalian taxa has been highlighted to ensure animal welfare is not compromised [10,37,38]. Species from non-primate taxa have different ecological backgrounds, behavioural repertoires, levels of cognition and relationships to zoo visitors. Quieroz and Young [39] emphasised specific ‘risk factors’ for zoo animals, principally suggesting that species which are terrestrial, herbivorous/omnivorous, have diurnal activity patterns and are from closed habitats are most likely to be at risk of experiencing negative impacts from zoo visitors. This covers many non-primate species, in particular animals considered to be prey species. These types of species may also be housed in different enclosures within zoos (e.g., in walk-through exhibits), which could impact their experiences of visitors and change the zoo visitor–animal dynamic [40].

A review by Sherwen & Hemsworth [5] collated a large amount of literature in relation to HAIs in zoo animals. Whilst this work produced a beneficial review, it focused on the human–animal literature as a whole, including a large amount of primate-specific literature yet did not add specific detail on the types of measures used in non-primate taxa. Lack of standardisation of methodologies designed to assess the impact of zoo visitors on animal experiences is a potential limitation for advancement in this field. Thus, compiling known information on methods used to understand the visitor effect in non-primate species is important.

Since the review by Sherwen & Hemsworth [5] there have also been a number of new visitor effect studies. These pieces of work have covered a range of taxa, including those which are typically understudied. The facility closures during the COVID-19 pandemic enabled the opportunity to investigate the visitor effect in terms of the presence/absence of zoo visitors (and absence of their other related stimuli, such as ambient background noise, even when the visitors are viewing other enclosures), which was limited previously to facilities that closed for periods of time over winter when other factors may be affecting animals.

An updated review which summarises the impact of visitors specifically on non-primate species within zoological collections, and identification of trends across taxa, is thus an important step in identifying knowledge gaps and helping to advance knowledge on the visitor effect more widely. This paper will quantitatively collate the literature on the visitor effect in non-primate species and investigate the types of measures used to assess that impact and consider whether these impacts varied across non-primate species in zoos. In doing so, we particularly sought to understand: (i) do measures differ by taxa? (ii) are there taxa-specific differences in terms of positive, negative or neutral responses to human visitors? and (iii) how do responses differ in relation to visitor characteristics? A secondary aim was to collate the papers published to date on the visitor effect in non-primate taxa, to act as a starting point for other researchers wishing to advance scientific knowledge in this area.

## 2. Materials and Methods

### 2.1. Search Methods

#### 2.1.1. Search Terms and Databases

A rapid review was undertaken in September 2022, with the cut-off date for papers being included in the review being 30 September 2022. To simplify the search, capturing a range of relevant papers without being exhaustive, the following combinations of search terms were used on Web of Science, Scopus and ProQuest: Visitor effect* AND zoos OR aquarium; Human–animal interaction* AND zoos OR aquarium; Animal–visitor interaction* AND zoos OR aquarium; Human–animal relationship* AND zoos OR aquarium; Animal–visitor relationship* AND zoos OR aquarium. Search terms were chosen based on terminology used by other researchers in this field, in order to maximise the capture of relevant records. Searches were restricted to title, abstract and keywords. In addition to this, to ensure papers were captured from zoo-specific journals which were not necessarily listed on these platforms, the same search terms were also entered into the Zoo Biology, Journal of Zoo and Aquariums Research (JZAR) and Journal of Zoological and Botanical Gardens (JZBG) sites. An additional search on Google scholar was conducted simply using ‘zoo visitor effect’ in order to maximise the likelihood of capturing all relevant literature. For database and specific journal searches, all papers were extracted. For Google Scholar, only the first 65 pages were searched. At this point, no more additional relevant papers were identified. All records were downloaded, and duplicates were removed manually.

#### 2.1.2. Inclusion/Exclusion Criteria

The aim of this work was specifically to capture papers which were focusing on the impact of zoo visitors on non-primate species. Owing to the recognition of the potential for animals to respond differently to familiar or non-familiar individuals [5], it was decided to focus this search only on visitors to zoos, where the presumption was that individuals are unknown to the animals and animals have not forged specific relationships or bonds with them. The following inclusion/exclusion criteria were applied to ensure only relevant papers were retained in the final review: (i) the paper must be based on or contain information on at least one non-primate species living in a zoological collection, (ii) the paper must be studying the effect of visitors on that species in a measurable context (n.b. this can include the presence/activity of visitors and also animal–visitor encounters, where visitors are engaged with interactions at closer proximities to animals, e.g., animal handling or animal feeding), (iii) the paper must be a primary research paper, (iv) the paper must be in a peer reviewed journal, (v) the paper must not be focusing on keeper–animal interactions, (vi) the paper must not be a conference proceedings or thesis (any stage).

**Figure 1:**
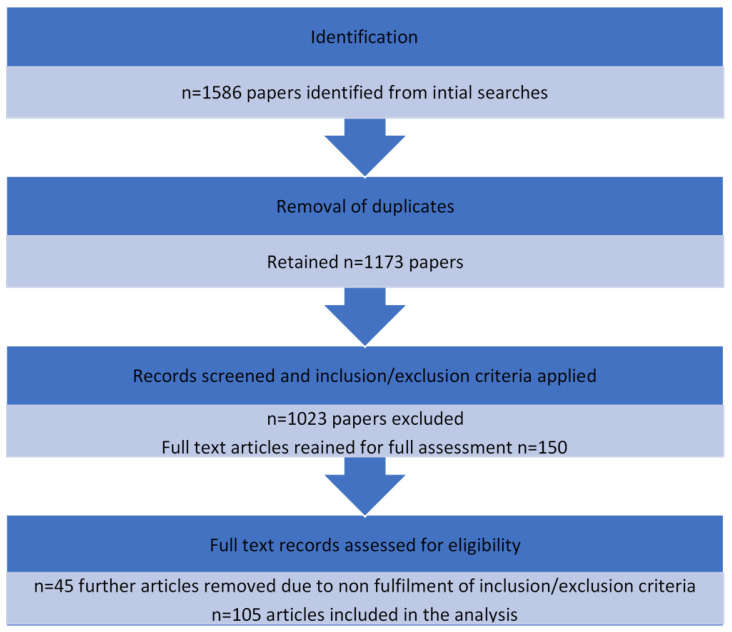
An overview of the process undertaken to identify and review relevant literature for the searches.

Initial searches identified 1586 potentially relevant papers. Removal of primate literature and other papers that did not completely meet inclusion and exclusion criteria left 105 studies suitable for inclusion in the final review (Figure 1).

#### 2.1.3. Data Extracted from the Identified Papers

The following information was pulled directly from the papers where relevant information was available: paper title, year of publication, non-primate species assessed, species order and family, location of the study, type of facility (e.g., zoo, safari park, aquarium), number of animals, methodology, visitor variables, enclosure details, animal-based measures assessed, change in animal-based measures, the valence of response to visitors reported by the authors if available. In two instances, species were reported together (housed in a mixed species enclosure and analysis was combined), these were treated as one species/one species group. The two groupings were two birds and two mammals (both of the same orders). If information on valence of response to visitors was not reported by the authors, then this was determined by EW based on animal-based measures. Where no clear valence was identifiable or where the authors reported difficulty in making any conclusive decisions, a category of ‘unknown’ was recorded.

### 2.2. Analysis

#### 2.2.1. Classification of Metrics

A selection of extracted metrics was classified to enable initial exploratory analysis and then basic inferential statistics. Types of animal-based measures used within each paper were classified as behavioural, physical or physiological. Where papers used more than one category of animal-based measure, these were added to all relevant categories (e.g., a paper measuring ‘behavioural’ and ‘physical’ measures was added to both the ‘behavioural’ and ‘physical’ category). Types of animal-based measures were also then classified at a species level within each paper, to enable the investigation of the relationship between this and the order and class of the species. Animal-based behavioural measures were then grouped according to whether the assessment involved detailed behavioural observations (i.e., full activity budgets) or simple observations (e.g., visibility of the animal). Finally, measures were categorised as being indicative of positive, negative or ‘unknown’ welfare state, to determine whether the animal-based measures were being used to capture positive or negative experiences. Changes in animal-based measures were classified as increasing, decreasing or no change. Visitor variables were grouped into nine key categories (presence/absence, events, interaction/handling programmes (e.g., education programmes or animal feeding experiences), noise, number, zoo open/closed, visitor access, visitor behaviour and visitor proximity). The valence of responses to visitors was categorised as positive, negative, no change or unknown.

#### 2.2.2. Statistical Analysis

Due to the categorical nature of the data, all assessments were undertaken using chi square tests, to document whether the findings differed from what would be expected by chance. A chi square goodness of fit test was used to investigate this in relation to: animal-based measures at a paper level (measures used by each paper), animal-based measures at a species level (measures used for each species within each paper), frequency of occurrence of visitor variables, frequency of detailed and simple behavioural measures, types of measures (positive, negative or unknown) and valence of the response to visitors (positive, negative, neutral, unknown). A chi square goodness of fit test was then used to investigate those measures in relation to taxa. Significance levels were set at 0.05 unless Bonferroni corrections were applied for multiple comparisons. Assessments undertaken were visitor variable frequency in relation to taxa, visitor variables and valence of response, frequency of animal-based measures by taxon, detailed and simple behavioural measures by taxon, type of measure by taxon, valence of response to visitors by taxon.

## 3. Results

### 3.1. Development of the Field

**Figure 2:**
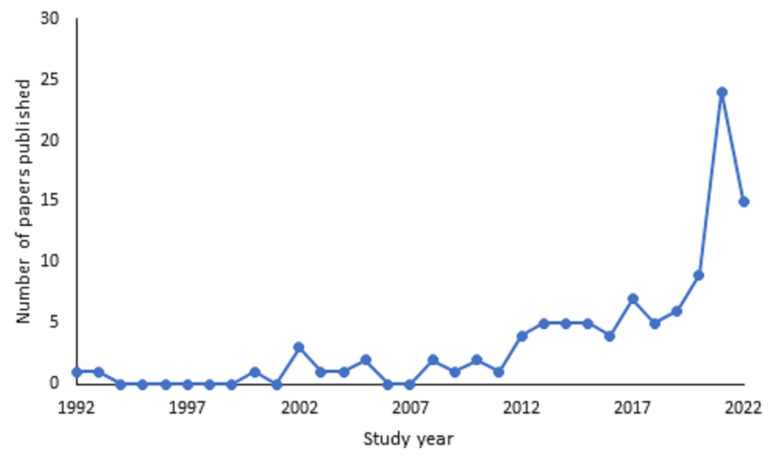
An overview of the number of papers produced per study year from 1992 to 2022.

Figure 2 shows that the earliest record of scientific papers on this topic were 1992 and 1993, with just one paper published each year. From 2000, there has been a steadier flow with a marked increase from 2013 onwards. There was a spike in 2021, when 24 papers were published; however, it is important to note that this upward trend may continue into 2022, as the review only captured papers published during the first 9 months of the year. This coincided with an increase in papers investigating the impact of the COVID-19 pandemic on zoo animals. Of the papers reviewed, 11% (n = 12) were focused on investigating the impact of the COVID-19 pandemic. In total, there were 105 separate papers included in the review, with 106 studies reported. One paper had two studies within it which was analysed as two separate studies due to the collection of different measurements in the second study. The majority of studies were opportunistic (n = 92, 87%), 11 papers (10%) were experimental (e.g., controls were put in place and modifications were made to enclosures or enclosure surroundings). Two papers combined both opportunistic and experimental methods.

**Figure 3:**
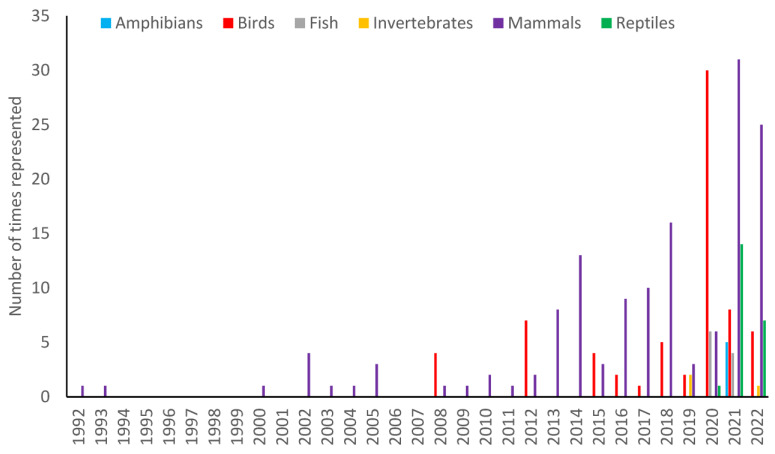
An overview of taxa represented in the study papers from 1992 to 2022.

Across the 105 research papers there were 252 animal species/species groups studied. The majority of the studied species were mammals (56%) and birds (28%). However, amphibians, reptiles, fish and invertebrates had all been studied to a greater or lesser extent. The diversification in species increased with the year of observation. Up until 2008, there were only mammalian species in the literature with the exception of one bird paper in 1992. There was then a gap in the study of birds until 2008. It is only since 2015 that birds have been consistently present. Invertebrates were not reported in the literature until 2019 and fish, reptiles and amphibians were not recorded until 2020/2021 (Figure 3).

**Figure 4:**
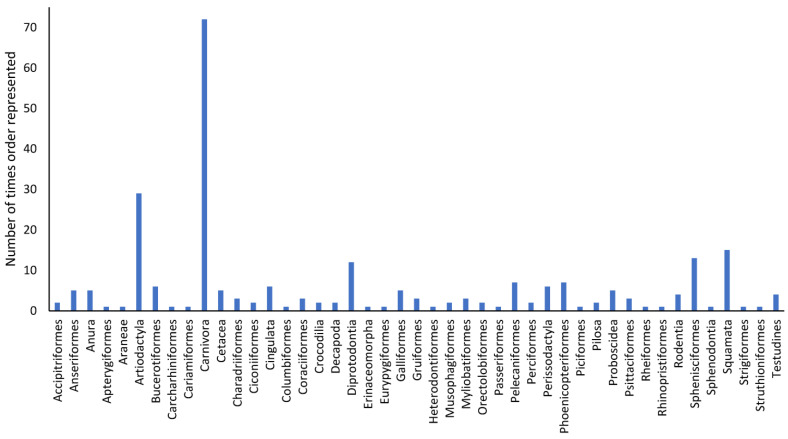
An overview of the orders represented in papers published on the visitor effect on zoo animals from 1992 to 2022.

There were 45 separate orders (Figure 4). Birds were represented by the most orders (n = 22 orders), followed by mammals (n = 10 orders). Fish were represented by n = 6 orders, reptiles by n = 4 orders, invertebrates by n = 2 orders and amphibians by n = 1 order. Carnivora were the most frequently represented order (n = 72), followed by Artiodactyla (n = 29). Diprotodontia, Sphenisciformes and Squamata were reported in n = 11, n = 13 and n = 15 instances, respectively. Anseriformes, Bucerotiformes, Cetacea (we have considered these separately because of different husbandry requirements, even though they are technically artiodactyls), Cingulata, Galliformes, Pelecaniformes, Phoenicopteriformes and Proboscidea were each represented from five to seven times. All other orders were recorded in less than five instances.

### 3.2. Visitor Variables

**Figure 5:**
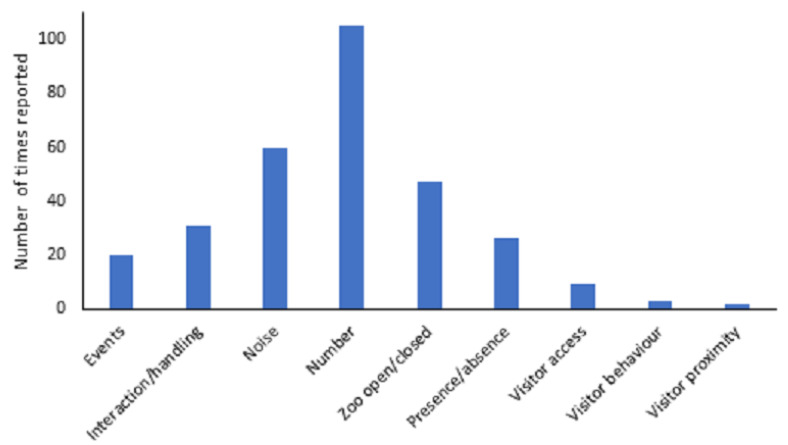
Range of visitor variables and number of times they were studied in the reviewed papers.

Visitor variables were categorised into nine broad themes: presence/absence, events, interaction/handling programmes (e.g., education programmes or animal feeding experiences), noise, number, zoo open/closed, visitor access, visitor behaviour and visitor proximity (Figure 5). Number (n = 105), noise (n = 60), and the zoo being open or closed (n = 47) were the most frequent visitor variables analysed in the studies. The frequency with which these were reported differed from what would be expected by chance (χ2 = 260.317, df = 8, *p* < 0.001). Noise, number and the zoo being open or closed were greater than would be expected by chance, whilst presence/absence of visitors, events, interaction/handling, visitor access, visitor behaviour and visitor proximity were all reported less than would be expected by chance.

The frequency with which these different visitor variables were assessed differed across taxa (χ2 = 245.108, df = 40, *p* < 0.001). Bonferroni corrected post hoc tests revealed presence/absence of zoo visitors to be greater than expected in amphibians (χ2 = 54.76, *p* < 0.0009); visitor access and response to interaction/handling were higher than expected in fish (χ2 = 49.00, *p* < 0.0009 and χ2 = 17.64, *p* < 0.0009, respectively); number of people was higher than expected in birds (χ2 = 12.25, *p* < 0.0009) and response to the zoo being open/closed were higher than expected in reptiles (χ2 = 102.01, *p* < 0.0009).

There was also a significant variation from what would be expected by chance in valence (positive, negative, neutral, unknown) and the visitor variable measured (χ2 = 54.438, df = 24, *p* < 0.001); however, these differences were not retained when Bonferroni corrected post hoc tests were undertaken (*p* > 0.001).

### 3.3. Animal-Based Measures

Response variables were broadly categorised into 35 categories. The majority of studies assessed behaviour (n = 95). Physiological variables were reported in 26 studies and physical measures in just one. Behaviour only measures were utilised in 80 studies and physiology only measures were used in 10 studies. A total of 15 used a combination of both behavioural and physiological variables and one paper used a combination of physiological and physical variables. Across the study papers, at an individual study level (i.e., variables used were summarised for the whole paper,) behavioural variables were greater than expected by chance, and physiological and physical variables were less than expected by chance (χ2 = 116.629, df = 2, *p* < 0.001).

**Figure 6:**
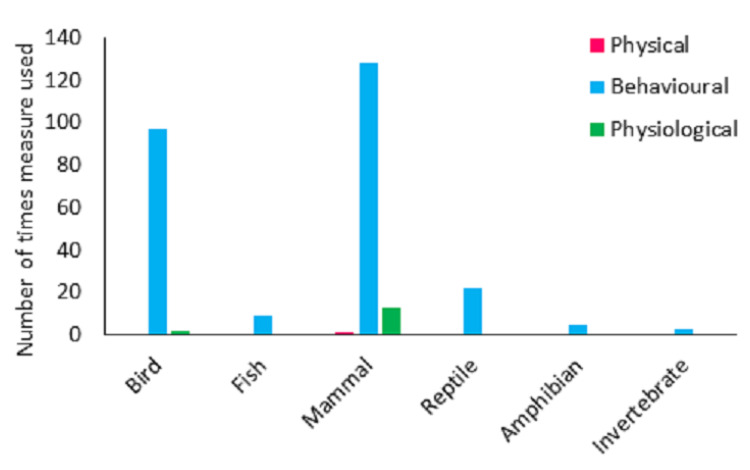
A breakdown of the number of behavioural, physical and physiological animal-based measures and how they varied across the animal groups.

The majority of papers (n = 71, 67.0%) assessed only one species. Fourteen papers (13.2%) assessed two species. The remaining papers were assessing three or more species. Across all of the species studied there were 280 variables assessed (n = 264 behavioural, n = 15 physiological and n = 1 physical). Some species were assessed using more than one method. Behavioural measures were greater than expected by chance and both physical and physiological measures were lower than expected by chance (χ2 = 469.164, df = 2, *p* < 0.001). The frequency of behavioural, physical or physiological variables did not however differ from what would be expected by chance across the taxa (χ2 = 9.479, df = 10, *p* = 0.487). Behavioural, physical and physiological measures were used in relation to mammals, behavioural and physiological measures were used in relation to birds. For reptiles, amphibians, fish and invertebrates, only behavioural observations were used (Figure 6).

Behavioural measures were separated into ‘detailed’ and ‘simple’ measures. Simple measures were those that could be undertaken relatively quickly and captured basic information, such as whether animals were visible or not, or where in the enclosure they were; detailed measures were those that represented fuller activity budgets or recorded specific behaviours. Across all taxa, detailed behavioural measures (n = 225 species) were greater than expected, and ‘simple’ measures were less than expected (n = 39 species) (χ2 = 130.045, df = 1, *p* < 0.001). This also varied across taxa (χ2 = 83.156, df = 5, *p* < 0.001). In amphibians and reptiles, simple assessments were more frequent than would be expected by chance (χ2 = 29.16, *p* < 0.001 and χ2 = 37.21, *p* < 0.001, respectively). In mammals, detailed assessments were more frequent than would be expected by chance (χ2 = 23.04, *p* < 0.001).

Of the 105 papers, there were 1795 separate animal-based measures (total number of single measures per paper, including where measures were used more than once to compare against multiple visitor variables) which were broadly categorised into 35 different types (Table 1), with some papers using different combinations for different species or different combinations for different aspects of the report. A total of 22 species within 12 papers had animal-based measures repeated for different visitor characteristics (e.g., the same animal-based measures to assess number of visitors and visitor presence/absence). Although recording the metrics each time they were used led to an inflation of the number of times each metric was used, this was considered most appropriate to capture the number of times each measure was used per study per visitor variable, and thus understand how the metrics were used in order to understand the impact of visitors in a range of different forms.

#### Animal-Based Measures by Taxa

There was a variation in type of animal-based behavioural measures across the taxa (Table 1). All but the ‘solitary’ and a generic ‘species-typical behaviour’ category were recorded for mammals, resulting in a total of 38 different measures. Across all taxa the most frequently used measures were: activity/inactivity (7.5%), enclosure use (9.2%), feeding (6.0%), locomotion (5.7%), positive social interactions (6.4%), resting (10.4%), stereotypical/abnormal behaviour (9.1%) and vigilance (5.1%). There were 27 separate measures for birds and 16 different measures for reptiles. There were eight separate metrics to assess the impact of visitors for fish although the majority of studies focused on five measures. There was only one amphibian study which focused on visibility and two invertebrate studies which encompassed five measures.

### 3.4. Categorisation of Animal-Based Indicators

Across the 105 studies, there were 1417 unique different animal-based indicators (i.e., number of indicators following removal of duplicates for assessment of different visitor measures). The majority (n = 647) were positive. A total of 345 indicators were classified as ‘unknown’ and 283 indicators were classified as ‘negative’. Enclosure use/proximity to visitors were used in 142 instances. This differed from what would be expected by chance (χ2 = 383.669, df = 3, *p* < 0.001). Positive animal-based indicators (e.g., positive social interactions, species-typical behaviour, engagement with the environment) were greater than expected by chance, negative animal-based indicators (e.g., stereotypies/abnormal behaviour, negative social interactions and vigilance) and enclosure use were less than expected by chance, and indicators classified as ‘unknown’ were as would be expected by chance. This varied across the different taxa (χ2 = 148.493, df = 15, *p* < 0.001). Bonferroni corrected post hoc tests showed that amphibian indicators were unknown more frequently than would be expected by chance (χ2 = 15.21, *p* < 0.002), enclosure use was more frequent than would be expected by chance in birds (χ2 = 10.89, *p* < 0.002) and fish (χ2 = 17.64, *p* < 0.002). Unknown indicators were more frequently reported in fish than would be expected by chance (χ2 = 12.96, *p* < 0.002). In mammals, positive (χ2 = 9.61, *p* < 0.002) and negative indicators (χ2 = 18.49, *p* < 0.002) were more frequent than expected by chance, whilst enclosure use (χ2 = 23.04, *p* < 0.002) and unknown indicators (χ2 = 17.64, *p* < 0.002) were less than expected by chance. In reptiles, positive (χ2 = 16.81, *p* < 0.002) and negative (χ2 = 18.49, *p* < 0.002) indicators were less than expected by chance, whilst unknown measures were more than expected by chance (χ2 = 70.56, *p* < 0.002).

### 3.5. Impact of Zoo Visitors on Animal-Based Measures

**Figure 7:**
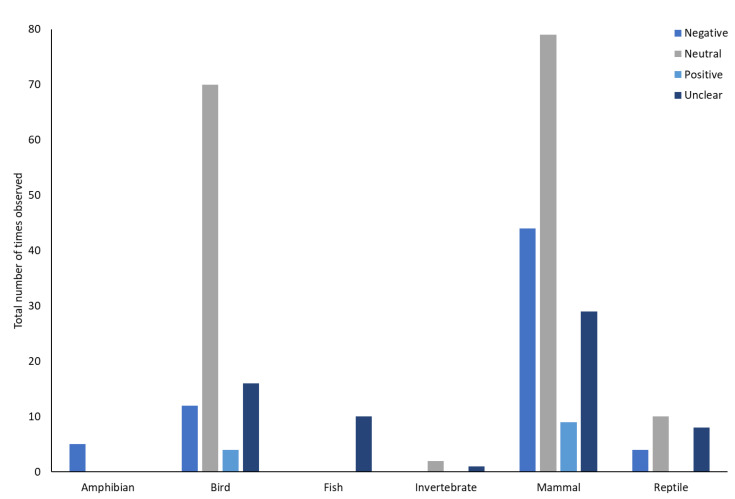
An overview of the number of times different responses were reported to zoo visitors across the taxa.

One paper was removed from the assessment of impact of visitors on animals as impacts were not reported in relation to individual species, rather specific ‘types’ of animals were considered as at greater or lesser risk of the impact of visitors. Across the remaining 104 studies there were 302 separate interpretations of the impact of visitors on the animals. The majority of these were neutral (n = 161), negative (n = 64) and unclear/could not be reliably stated from the study (n = 64). Positive impacts were only reported in 13 cases. Valence across all of the studied taxa differed from what would be expected by chance (χ2 = 152.066, df = 3, *p* < 0.001). Negative, positive and ‘unclear’ were all less than expected by chance. Neutral responses to visitors were greater than would be expected by chance. The valence of the impact of visitors varied across taxa (χ2 = 75.062, df = 15, *p* < 0.001, Figure 7). Bonferroni corrected post hoc tests revealed that amphibians had negative responses more frequently than would be expected by chance (χ2 = 18.49, *p* < 0.001), birds (χ2 = 14.44, *p* < 0.002) and fish (χ2 = 11.56, *p* < 0.001) had neutral responses more than expected by chance and ‘unclear’ responses were reported more frequently than expected by chance in fish (χ2 = 38.44, *p* < 0.002). There were no differences from what would be expected by chance for positive, neutral or unclear responses in amphibians, positive, negative or unclear responses in birds and positive or negative responses in fish (*p* > 0.05). For invertebrates and mammals there were no differences from what would be expected by chance in any of the categories (*p* > 0.05).

Across all of the orders, the most frequent response was neutral (n = 26 orders), and the least frequent response was positive (n = 2 orders). Negative responses were most frequently recorded in n = 10 orders and ‘unknown’ responses were recorded most frequently in n = 14. Valence of response to visitors varied across orders (χ2 = 244.259, df = 132, *p* < 0.001, Table 2). Bonferroni corrected post hoc tests suggested the differences were in Anura, who had negative responses to visitors more frequently than would be expected by chance (χ2 = 18.49, *p* < 0.0002), Proboscidea, who had positive responses to visitors more frequently than would be expected by chance (χ2 = 38.44, *p* < 0.0002) and Psittaciformes who also had more positive responses to visitors than would be expected by chance (χ2 = 28.09, *p* < 0.0002).

With the exception of ‘enclosure use’, indicator changes were classified as ‘increased’, ‘decreased’ or ‘no change’. ‘Enclosure use’ was classified as those main categories, plus ‘moved further from visitors’, ‘moved closer to visitors’ or just ‘changed’ if it was not clear how this change related to the location of visitors or the enclosure in general. For the majority of animal-based measures, there was ‘no change’. Only three increased more frequently than they decreased or did not change in response to visitors: solitary behaviour (n = 1); cortisol/corticosteroids (n = 16) and behavioural diversity (n = 3). Abrupt behaviour (n = 1) and proximity to visitors (n = 5) decreased more frequently than they increased or did not change. For all of the other measures, the most frequent response was ‘no change’ (Table 3). A breakdown of behaviour change by taxa and an overview of the most studied families within each taxon are included in the supplementary material (Files S1 and S2).

## 4. Discussion

The aim of this work was to quantify the impact of zoo visitors on non-primate species, and to understand whether this replicated what was known from the zoo primate literature or whether differences are seen amongst other non-primate mammals or other species.

### 4.1. Species Studied

As with other zoo research, the majority of studies were on mammals. However, in recent years, more work has been undertaken on other zoo housed animals, including invertebrates, fish, reptiles, amphibians and birds. This expansion into the assessment of lesser-studied taxa is important for ensuring animal welfare in a range of species. However, whilst research has been expanded into these taxa, this review indicates that the number of studies remains minimal for some taxa. The need to consider the impact of zoo visitors on all animals has been highlighted [38] and this research supports that assertion.

### 4.2. Animal-Based Measures Used to Assess Impacts of Visitors

Across all taxa, the most frequently represented measures were activity/inactivity, enclosure use, feeding, locomotion, positive social, resting, stereotypical/abnormal and vigilance behaviour. These behaviours changed in 17 to 38% of cases. Breathing rate, bodyweight, heart rate and cortisol/corticosteroids were also used but far more rarely, although where these were used, the percentage of times they were reported to change was higher (breathing rate: 50%; cortisol: 52%; heart rate: 50%; bodyweight: 100%) than other indicators. Indicators used varied across taxa with mammals having the most separate indicators used for them. Measures that changed also varied across taxa. For amphibians, the only measure that was used was visibility, this did change in relation to visitors. In invertebrates, enclosure use, and grooming changed in all studies. For fish, reptiles, birds and mammals, there were more measures used and more measures that changed, so only those that changed in more than 30% of cases are detailed here. Four measures changed in fish: enclosure use, resting, swimming and solitary behaviour. In reptiles, six measures changed: activity/inactivity, behavioural diversity, negative social, positive social, proximity to conspecifics and visibility. Ten measures changed in birds: behavioural diversity, maintenance behaviours, proximity to conspecifics, proximity to visitors, HAIs (negative and positive), resting, swimming, vigilance and cortisol. In mammals, there were 15 measures which changed: abrupt behaviour, activity/inactivity, behavioural diversity, enclosure use, HAIs, proximity to conspecifics, proximity to visitors, reactivity to visitors, stationary, swimming, visibility, heart rate, breathing rate, cortisol and bodyweight. The majority of animal-based indicators were indicative of positive welfare state. This is representative of the development of the field of zoo animal welfare science and the shift of focus from indicators of negative experiences in zoo animals to indicators of positive experiences [41].

Studies used both simplistic and detailed approaches to capturing data. In mammals, detailed assessments were more frequent, whilst in amphibians and reptiles, simplistic assessments were more frequent. This may reflect the activity level of the species being observed with simpler measures being used for species who are not normally as active or are more cryptic and therefore harder to observe. Or it could reflect the fact that for mammalian species more research has been conducted to identify key welfare indicators and these were being incorporated for these species. There is the potential for studies only using simplistic measures to miss the high-level detail required to ascertain whether visitors were negatively impacting on species [42], but snapshot measurements which give an overview of what animals are doing have been advocated for use in zoos [43]. Thus, it might be beneficial to spend longer looking at some species to enable an understanding of type of activity, but doing this in a snapshot fashion which enables this assessment method to fit into zoo routines.

Sherwen and Hemsworth [5] highlighted the importance of understanding what the measures used are showing in relation to animal welfare and Meade et al. [44] suggested there should be greater incorporation of metrics which relate to the assessment of animal welfare and emotional experiences of animals when investigating the impacts of HAIs. However, using measures which are likely to capture changes in animal states in relation to humans is also important to ensure the metrics used to assess their welfare are reliably rating what they are supposed to be rating.

It is recommended that researchers incorporate a suite of different animal-based measures and in doing so capture the combined impact of conditions [9]. Specifically, (i) measures should be meaningful in terms of being representative of individual animal experiences and animal welfare and (ii) methods used should capture those behaviours accurately. Measures should thus depend upon the study species. This review has highlighted the range of measures used for different taxa and highlighted those that changed most frequently within the literature. The fact that these measures have changed in previous work suggests they may be appropriate measures moving forwards. However, experimental work should confirm this, using a suite of indicators to capture all aspects of the impact of visitors on species.

### 4.3. Impacts of Zoo Visitors on Non-Primate Species: Differences across Taxa and Orders

Across all of the study papers there were 302 interpretations of the impact of visitors. 53% were neutral, 21% were negative, 21% were ‘unclear’ and only 4% were considered to be positive. Differences were seen across taxa. Amphibians were more likely to be negatively affected by the presence of zoo visitors, birds had neutral responses to visitors and fish were predominantly classified as having neutral responses or the responses being unknown. Sixteen orders showed at least some negative responses to visitors, whilst only six showed some positive responses. Specifically, at the level of orders, Anura had negative responses more frequently than was expected and Proboscidea and Psittaciformes had positive responses more frequently than would be expected. Queiroz & Young [39] highlighted risk factors in relation to behavioural ecology of species, stating that prey species that were from closed habitats (e.g., forests) or had nocturnal activity patterns, where they were less likely to encounter humans, may make animals more fearful of humans. However, many animal species within zoos have been born and raised in zoos, and so have likely become habituated to the presence of humans [45]. Zoo animals also show adaptability to the changing conditions of visitors in zoos [15]. Despite this, some of the species in this study matched with the predictions made by Queiroz & Young [39]. Animal groups for whom visitors were reported to have a negative impact included flightless birds, odd and even-toed ungulates, marsupials, ostriches, tuatara and hedgehogs. There were also some species who may typically be considered to be more cryptic in their behaviour, such as scaled reptiles and frogs. For all of those species, visitors could potentially be perceived as a threat.

Species of Carnivora showed positive, negative and neutral responses to zoo visitors. Members of this order are usually predatory species and thus this finding does not match with the predictions made by Quieroz and Young, that predatory species are naturally more aggressive and therefore less likely to be threatened by the presence of visitors [39]. However, as well as being largely predatory species, there is also a high number of charismatic species (e.g., lions (*Panthera leo*), tigers (*Panthera tigris*), leopards (*Panthera pardus*)) or species that are typically of interest to zoo visitors (e.g., meerkats (*Suricata suricatta*)) in the order Carnivora and so it is possible that the range of responses was related to other factors, including visitor relationships with animals and the type of interaction visitors were having with the animals (e.g., in ambassador programmes), number of visitors at enclosures or visitor behaviour towards the animals. Mammals have greater attractive properties and holding time than other species in zoos and flagship species in particular draw the attention of zoo visitors [12]. Animals in the order Carnivora therefore could have been exposed to greater levels of ‘stress’ caused by visitors, which may have led to the variable responses observed in the reviewed studies.

Proboscidea and Psittaciformes were the only two orders who displayed positive responses to visitors more frequently than would be expected by chance. Proboscidea also displayed neutral responses but no negative responses. Psittaciformes also displayed negative responses. For both of these orders, there were only a small number of studies representing them, so the results must be interpreted with caution. It is unclear what the cause may be. In primate species, animals with a smaller body size are more likely to be negatively affected by the presence of zoo visitors (Hosey et al., in review). It was beyond the scope of this review to assess the order data at the level of individual species size, owing to the variability of some order categories; however, Proboscidea are the largest land mammals, and so this could have led to a reduced effect of zoo visitors. It would be expected that zoo visitors would be drawn to elephants, as a large flagship species [12]. However, the reduced impact of visitors in this work could be due to enclosure design. As a dangerous animal, elephants need to be kept safely separated from the public, which may reduce the opportunity for human behaviours which negatively impact on elephant experiences. Parrots are highly intelligent birds who are known for their need for interaction and attention, and for their desire to interact with visitors in zoo environments [46]. The positive valence recorded in this review could thus be a product of birds trying to gain attention from and seeking interactions with zoo visitors, as was recorded during the COVID-19 pandemic [47].

As with Proboscidea and Psittaciformes, the results on valence of visitors in relation to other orders must be interpreted with caution. Due to the relatively small numbers of study animals for some of the orders it is not possible to extrapolate these data to state with certainty that zoo visitors will have positive, negative or neutral effects on all species within these orders in all collections. It is rather more likely, as has been highlighted previously, that there will be variable effects as a result of inter-individual differences or husbandry or enclosure design factors. Indeed, individual variability was reported within some of the studies, which emphasises the importance of understanding animal experiences at an individual level. This is similar to what has been concluded for primate species, which indicates large levels of individual variability in response to zoo visitors. There was also disparity in how varied the orders were. Some were representing rather narrow species ranges (e.g., Proboscidea) whilst others were much more variable, incorporating numerous species (e.g., Carnivora), which means that even within orders, there is likely to be a range of ‘risk’ levels for different species, with species being at a greater or lesser risk of being affected by visitors.

### 4.4. Visitor Variables and the Potential for Differential Impacts

As has been highlighted previously [5,27] and throughout this review, the relationship between zoo animals and zoo visitors is complex. Although there was no association between behavioural responses to visitors and the visitor metrics recorded, it is probably not just the presence of zoo visitors that impacts on animals, rather it is more likely to also relate to visitor activity and behaviour. Active animals can lead to higher levels of visitor interaction and activity [25]. In little penguins (*Eudyptula minor*), visitor activity appears to affect animal behaviour, and when visitor access to the enclosure was controlled a reduced impact of the presence of visitors was seen [48]. Similar findings have been reported in tourism destinations as well, with ‘active’ human presence triggering the greatest vigilance responses in the birds [49]. Within primate species incidences where visitors are trying to interact with zoo animals seem to have the greatest effects on animal behaviour, particularly when visitors appear larger, are louder or more aggressive, or are making repeated attempts to make contact with the animal [6]. These responses to ‘active’ people correspond with the theory of risk of predation, proposed by Quieroz & Young [39]. However, reducing the threat of visitors can be achieved through experimental design, such as provision of hidden areas or by reducing the perimeter of enclosures which visitors can access, both of which were modifications in the reviewed papers which led to a reduction in metrics indicative of negative experiences of visitors [48,50,51,52,53].

It can be difficult to disentangle ‘the visitor effect’ from other factors which could be influencing zoo animal response to visitors—e.g., impacts of time of year, social relationships, management changes or other aspects of visitors beyond those in which you are actively assessing (i.e., assessing the impact of visitor interaction programmes but not knowing whether changes recorded are related to the presence of visitors or alteration of routines). Taking an experimental approach to HAI research may help to reduce or eliminate this, such as focusing on controlling different aspects of the study, repeating assessment at different times of year, and repeating assessments for individuals in different conditions to capture individual variation, etc.

### 4.5. Limitations and Future Directions

Behaviours were only recorded as ‘changed’ if significant differences in behaviour frequency were reported. This may have led to an underestimation of some behavioural changes which could signify biologically relevant impacts on animals. For example, an increase in stereotypical behaviour was only recorded if that increase was statistically significant and was recorded in the results section of the reviewed papers. For understanding the impacts on animals, it may be better to acknowledge that any increase in stereotypies would be relevant, regardless of whether or not statistical significance was reached. However, only including statistically significant changes enabled consistency across the papers, as it is possible that some papers just did not report any non-significant changes in behaviour, owing to authors not considering it a relevant finding.

Wherever possible the author interpretations of animal experiences of visitors were used, rather than interpreting the valence of behavioural change. This was performed in acknowledgment of the fact that there may be more to the interpretation of behaviour than simply measuring a change in one metric [5], and that authors undertaking the research were likely to have a better all-round interpretation of the impact of visitors on the study animals, taking into account potentially smaller or less obvious changes and capturing the nuances of these sometimes complex interactions. However, there is the potential for there to be bias or hesitation in interpretation/reporting, which must be borne in mind during the interpretation of the results.

The impacts of individual animal experiences within the zoo have been highlighted [54] and the potential for this to affect experiences of visitors is also clearly recognised [5]. Where data were captured on an individual level in papers included in this review there were some individual differences seen. As not all papers analysed data on an individual basis, it could be that individual-level variation was masked by combined data. The fact that some individuals may be positively affected by zoo visitors whilst others are affected negatively by zoo visitors emphasises again the importance of considering zoo management on an individual animal basis wherever possible.

Controlled experimental setups might help to more clearly disentangle the range of factors which may impact on animal behaviour in zoos, from the true impact of visitors on animals. Understanding the impact of visitors in relation to specific (named) husbandry variables between collections will also be advantageous. Specifically, we recommend that more quantitative work is undertaken to measure the range of visitor characteristics in multiple venues, considering how different husbandry and visitor variables impact on different species, and developing metrics to reliably assess welfare and experiences for those animals.

## 5. Conclusions

The ‘visitor effect’ in zoos is a well-recognised phenomenon that has been particularly widely studied in primate species. In more recent years, this field has been advanced to start to include other mammalian species, and since 2008 it has been extended to non-mammalian taxa. This extension into non-primate species and non-mammalian species is beneficial to zoo animal welfare, but there is still a bias towards charismatic species. There has been a steady increase in visitor effect research in zoos since 2012. Although there has been an increase, the majority of work is opportunistic, looking at numbers of visitors and comparing that using correlational methods to animal behaviour. Controlled experimental setups might help to more clearly disentangle the range of factors which may impact on animal behaviour in zoos, from the true impact of visitors on animals. Understanding the impact of visitors in relation to specific (named) husbandry variables between collections will also be advantageous. It is recommended that moving forwards researchers incorporate a suite of measures, incorporating those which are meaningful in terms of being representative of individual animal experiences and animal welfare, collected in a manner which should capture those metrics accurately.

## Figures and Tables

**Table 1:** An overview of animal-based measures by animal category (total number per paper, including where measures were used more than once to compare against multiple visitor variables. Total number = 1795). The most frequent measures (accounting for >5% of total recordings for that animal type) are displayed in bold.

Animal-Based Measure	Category	Animal Category					
		Mammal	Bird	Reptile	Amphibian	Fish	Invertebrate
Abrupt behaviour	Behaviour	1	0	0	0	0	0
Activity/inactivity	Behaviour	**79**	**33**	**22**	0	1	0
Behavioural diversity	Behaviour	1	1	**6**	0	0	0
Breathing rate	Physiological	1	0	0	0	0	0
Bodyweight	Physical	2	0	0	0	0	0
Cortisol/corticosteroids	Physiological	27	6	0	0	0	0
Enclosure use	Behaviour	**57**	**83**	**11**	0	**14**	**1**
Feeding	Behaviour	**79**	**25**	3	0	0	0
Grooming	Behaviour	52	**35**	0	0	0	**1**
HAI	Behaviour	8	3	0	0	0	0
HAI negative	Behaviour	36	3	0	0	0	0
HAI positive	Behaviour	30	6	0	0	0	0
Heart rate	Physiological	2	0	0	0	0	0
Interaction with environment	Behaviour	43	18	**7**	0	**6**	**1**
Locomotion	Behaviour	**76**	23	3	0	0	**1**
Maintenance	Behaviour	2	1	1	0	0	0
Negative social	Behaviour	**59**	20	2	0	0	0
Nervous	Behaviour	2	0	0	0	0	0
Olfactory	Behaviour	16	0	0	0	0	0
OOS	Behaviour	53	9	**11**	0	0	0
Other	Behaviour	22	**57**	2	0	0	0
Positive social	Behaviour	**83**	21	**8**	0	2	0
Proximity to conspecifics	Behaviour	10	4	1	0	0	0
Proximity to visitors	Behaviour	10	2	0	0	0	0
Reactivity to people	Behaviour	1	0	0	0	0	0
Reproduction/maternal	Behaviour	14	10	0	0	0	0
Resting	Behaviour	**148**	**27**	2	0	**10**	0
Solitary behaviour	Behaviour	0	0	0	0	2	0
Species typical	Behaviour	0	2	0	0	0	0
Stationary	Behaviour	7	2	0	0	0	0
Stereotypical/abnormal	Behaviour	**96**	64	0	0	**4**	0
Swimming	Behaviour	7	13	0	0	**13**	0
Vigilance	Behaviour	**76**	15	1	0	0	0
Visibility	Behaviour	13	8	**16**	**5**	0	**2**
Vocalisation	Behaviour	19	12	1	0	0	0

**Table 2:** An overview of response to visitors as a function of animal order. The most frequent behaviour response is highlighted in bold for each animal-based measure.

Order	Taxa	Animal Type	Negative	Neutral	Positive	Unknown
Accipitriformes	Bird	Birds of prey	**1**	**1**	0	**1**
Anseriformes	Bird	Waterfowl	0	**8**	0	1
Anura	Amphibians	Frogs	**5**	0	0	0
Apterygiformes	Bird	Flightless birds	**2**	0	0	0
Araneae	Invertebrate	Spiders	0	0	0	**1**
Artiodactyla	Mammal	Even toed ungulates	9	**21**	1	2
Bucerotiformes	Bird	Hornbills and hoopoes	0	**4**	0	3
Cariamiformes	Bird	Flightless birds	0	**2**	0	0
Carcharhiniformes	Fish	Ground sharks	0	0	0	**1**
Carnivora	Mammal	Cat- and dog- like eutherian mammals	21	**43**	4	17
Cetacea *	Mammal	Whales, dolphins and porpoises	0	**3**	0	2
Charadriiformes	Birds	Shore birds	0	**2**	0	**2**
Ciconiiformes	Birds	Stork like birds	0	**3**	0	0
Cingulata	Mammal	Armadillos	**2**	0	0	1
Columbiformes	Birds	Pigeons and doves	0	**2**	0	0
Coraciiformes	Birds	Medium sized colourful birds	0	**6**	0	0
Crocodilia	Reptiles	Alligators, caimans, crocodiles, gharial and false gharial	0	**1**	0	**1**
Decapoda	Invertebrates	Crustaceans	0	**2**	0	0
Diprotodontia	Mammals	Marsupials	**8**	5	0	4
Eulipotyphla	Mammals	Insectivorous mammals	**1**	0	0	0
Eurypygiformes	Birds	Kagus and sunbittern	0	**2**	0	0
Galliformes	Birds	Chickens	0	**7**	0	1
Gruiformes	Birds	Crane-like birds	0	**5**	0	1
Heterodontiformes	Fish	Bullhead sharks	0	0	0	**1**
Musophagiformes	Birds	Turaco	0	**2**	0	1
Myliobatiformes	Fish	Stingray	0	0	0	**3**
Orectolobiformes	Fish	Carpet shark	0	0	0	**2**
Passeriformes	Birds	Passerines	0	0	0	1
Pelecaniformes	Birds	Large water fowl	0	**11**	0	0
Perciformes	Fish	Perch-like fish	0	0	0	2
Perissodactyla	Mammal	Odd-toed ungulate	**3**	**3**	0	0
Phoenicopteriformes	Bird	Flamingo	1	**5**	0	1
Piciformes	Bird	Woodpecker	0	0	0	**1**
Pilosa	Mammal	Anteaters and sloths	0	**1**	0	**1**
Proboscidea	Mammal	Elephants	0	2	**3**	0
Psittaciformes	Bird	Parrots	1	0	**2**	0
Rheiformes	Bird	Flightless ratite birds	0	**1**	0	0
Rhinopristiformes	Fish	Shark-like rays	0	0	0	**1**
Rodentia	Mammal	Rodents	0	1	1	**3**
Sphenisciformes	Bird	Penguin	**6**	**6**	2	2
Sphenodontia	Reptile	Tuatara	**1**	0	0	0
Squamata	Reptile	Lizards and snakes	2	**8**	0	5
Strigiformes	Birds	Owls	0	**2**	0	0
Struthioniformes	Birds	Ostriches	**1**	**1**	0	0
Testudines	Reptile	Turtles	1	1	0	**2**

* Cetacea are here listed as a separate order although technically they are artiodactyls.

**Table 3:** An overview of animal-based measures and the direction of the behaviour change. The most frequent behaviour change category is highlighted in bold for each animal-based measure.

Animal-Based Measures	Behaviour Change						
	Changed	Closer	Further	Decreased	Increased	None	% Changed
Abrupt behaviour	0	0	0	**1**	0	0	100
Activity/inactivity	0	0	0	27	24	**84**	38
Behavioural diversity	0	0	0	2	**3**	**3**	63
Breathing rate	0	0	0	0	1	1	50
Bodyweight	0	0	0	1	0	0	100
Cortisol/corticosteroids	0	0	0	1	**16**	**16**	52
Enclosure use	12	10	17	6	6	**115**	31
Feeding	0	0	0	10	11	**86**	20
Grooming	0	0	0	5	7	**76**	14
HAI	0	0	0	1	2	**8**	27
HAI negative	0	0	0	0	6	**33**	15
HAI positive	0	0	0	3	6	**27**	25
Heart rate	0	0	0	1	0	**1**	50
Interaction with environment	0	0	0	7	5	**63**	16
Locomotion	0	0	0	7	11	**85**	17
Maintenance	0	0	0	1	0	**3**	25
Negative social	0	0	0	4	7	**70**	14
Nervous	0	0	0	0	0	**2**	0
Olfactory	0	0	0	0	1	**15**	6
OOS	0	0	0	1	4	**68**	7
Other	0	0	0	1	2	**78**	4
Positive social	0	0	0	8	12	**94**	18
Proximity to conspecifics	0	0	0	4	3	**8**	47
Proximity to visitors	0	0	0	**5**	3	4	67
Reactivity to people	0	0	0	0	1	0	100
Reproduction/maternal	0	0	0	1	0	**23**	4
Resting	0	0	0	29	14	**144**	23
Solitary behaviour	0	0	0	0	**1**	**1**	50
Species typical	0	0	0	0	0	**2**	0
Stationary	0	0	0	1	2	**6**	33.3
Stereotypical/abnormal	0	0	0	12	16	**136**	17
Swimming	0	0	0	5	9	**19**	42
Vigilance	0	0	0	6	23	**63**	32
Visibility	0	0	0	16	4	**24**	45
Vocalisation	0	0	0	1	2	**29**	9

## Data Availability

Overview of reviewed papers is available at: https://tinyurl.com/yc68ppuw (accessed on 21 March 2023).

## Supplementary Materials

The following are available online at https://www.mdpi.com/article/10.3390/ani13071178/s1, File S1: A brief overview of the most studied families within each taxon; File S2: Details on the way in which each animal-based measure changed in the different taxa or animal categories (Tables S1–S6).
