# Supplementary material for: The Impact of Visitors on Non-Primate Species in Zoos: A Quantitative Review
Source: Animals (Basel). 2023 Mar 28;13(7):1178. doi: 10.3390/ani13071178 (PMC10092984; doi:10.3390/ani13071178)
Supplement: Supplementary file 1 [file animals-13-01178-s001.zip › Williams et al_supplementary material_S1.pdf]

Supplementary material

File 1: A brief overview of the most studied families within each taxon

Mammals

142 mammals were included in the reviewed papers. These spanned 10 orders and encompassed 103 separate species from 27 families. The most frequently represented were cheetah (*Acinonyx jubatus*) (n=5), jaguar (*Panthera once*) (n=5), tiger (*Panthera tigris*) (n=5), ocelot (*Leopardus pardalis*) (n=4), leopard (*Panthera pardus*) (n=4), lion (*Panthera leo*) (n=4), giraffe (*Giraffa camelopardalis*) (n=4), African elephants (*Loxodonta Africana*) (n=3), Giant panda (*Ailuropoda melanoleuca*) (n=3), meerkat (*Suricata suricatta*) (n=3), puma (n=3) and red kangaroo (*Macropus rufus*) (n=3). The most represented families (representing more than 5% of the mammals studied) were: Bovidae (9.2%), Felidae (26.8%), Macropodidae (7%), Ursidae (9.2%). Animal based measured across mammalian species were grouped into 25 different types of measure (Figure 1). Across all mammalian species the most commonly used animal-based indicators were positive social, locomotion, human-animal interactions, activity/inactivity, vigilance, stereotypical/abnormal and resting. An overview of response to different visitor characteristics at a family level (for Felidae, Bovidae, Macropodidae and Ursidae) is available online at https://tinyurl.com/yc68ppuw.

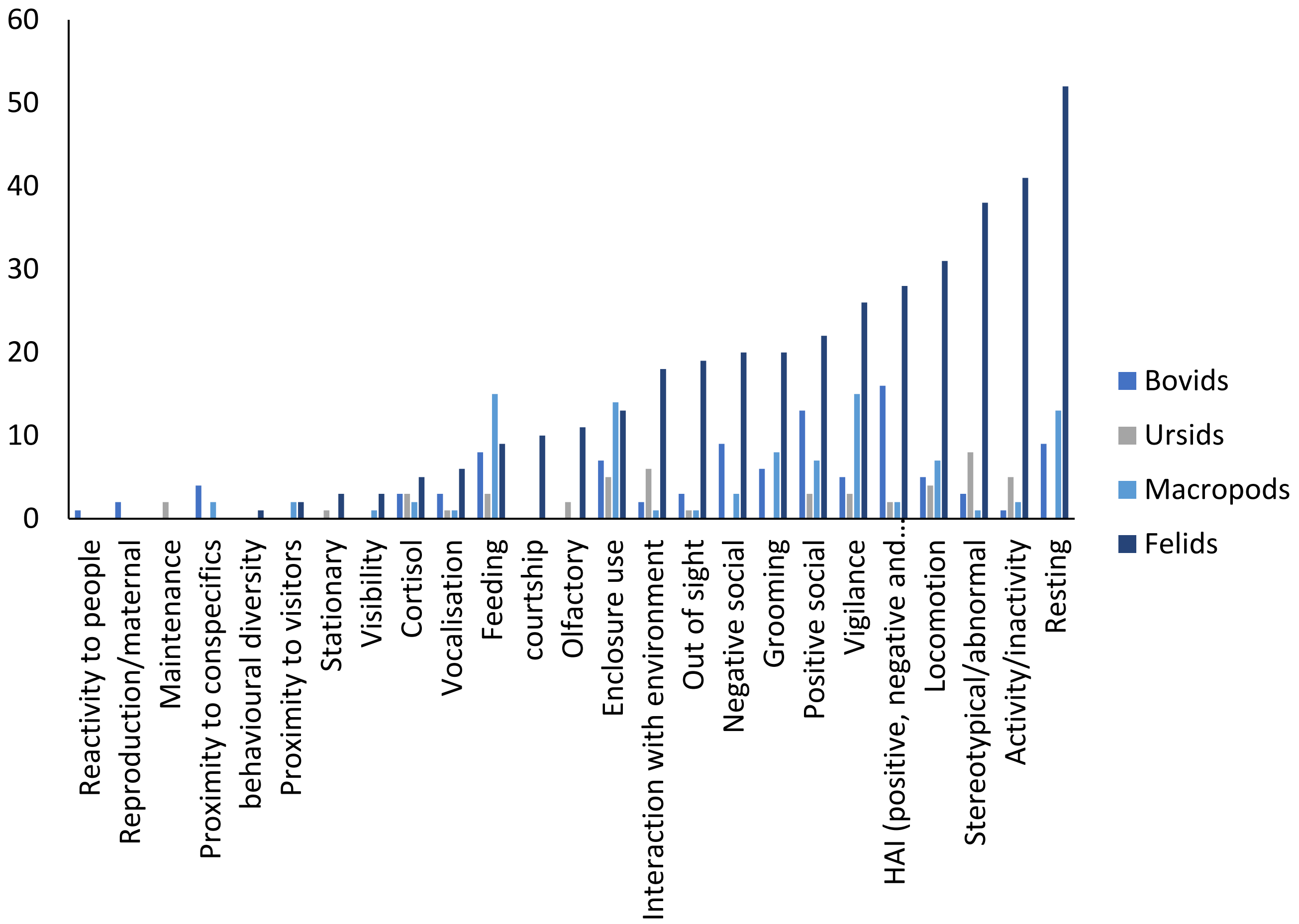

Figure S1. An overview of welfare indicators used across mammalian species

Felidae

Felidae were the most frequently studies species. 21 papers investigated the impact of visitors on at least one felid species with 48 different species/visitor variable/response to visitor combinations in total. In total there were 236 felids included in the reviewed papers. There were 13 species of felid: tiger, bobcat (*Lynx rufus*), Canada lynx (*Lynx canadensis*), cheetah, clouded leopard (*Neofelis nebulosa*), jaguar, leopard, lion, Eurasian lynx (*Lynx lynx*), ocelot, puma (*Puma concolor*), serval (*Leptailurus serval*) and snow leopard (*Panthera uncia*). The majority of responses to visitors were neutral (n=20), negative (n=13) and unclear (n=11). There were only four positive impacts reported. Number of visitors was the most frequently reported visitor variable (n=17). Noise and presence/absence of visitors were reported in n=10 instances each, response to events or interaction programmes in n=5 instances and visitor proximity in just one. The animal-based indicators most frequently used across the felids studied were: out of sight (5%), negative social (5.3%), grooming (5.3%), positive social (5.8%), vigilance (6.9%), human-animal interactions (positive, negative and uncategorised) (7.4%), locomotion (8.2%), stereotypical/abnormal behaviour (10.1%), activity/inactivity (10.8%) and resting (13.8%). The measures that changed most frequently (in terms of percentage of papers in which they were studied that the measure then changed) were human-animal interactions (33.3%), stationary (33.3%), vigilance (33.3%), resting (35.7%), stereotypies (41.9%), feeding (42.9%), enclosure use (44.4%), cortisol (60%), activity (61.5%), behavioural diversity (100%), proximity to visitors (100%) and visibility (100%) (Figure 2).

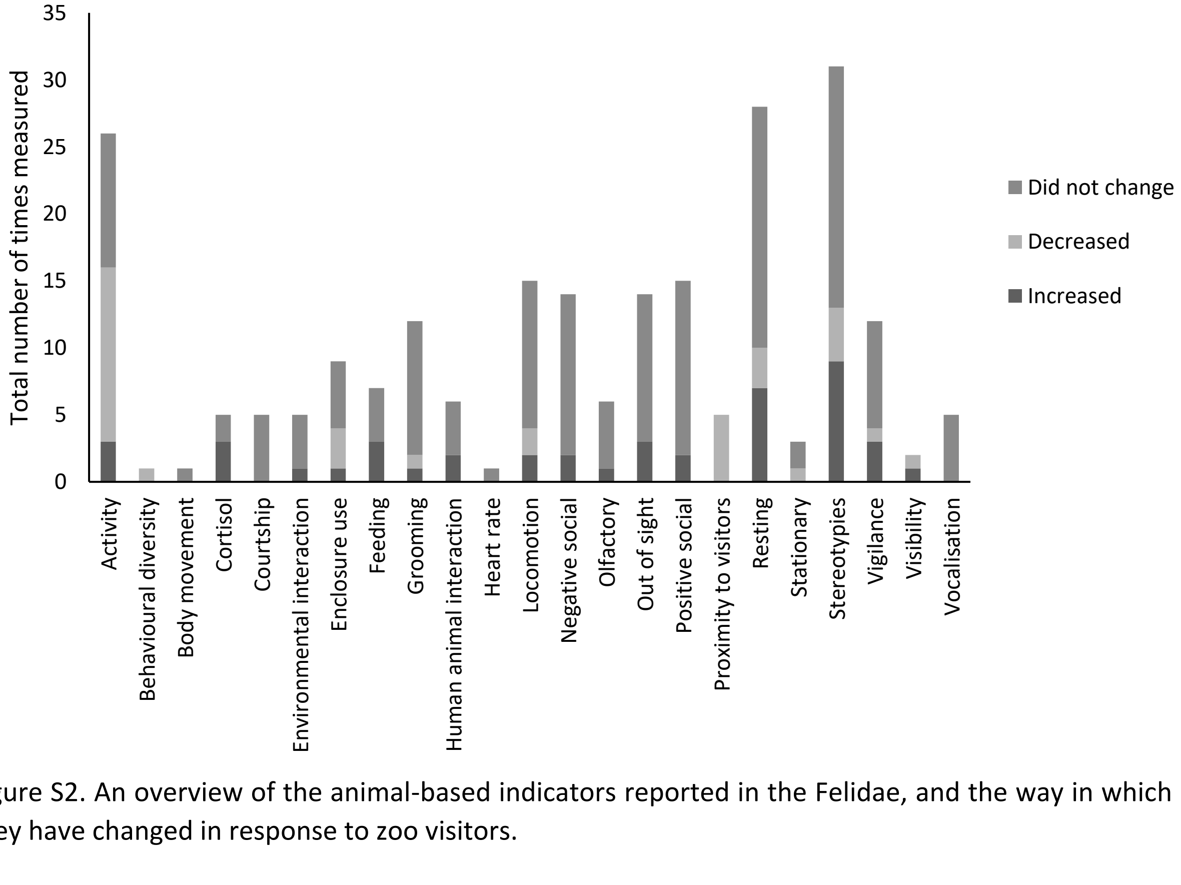

Figure S2. An overview of the animal-based indicators reported in the Felidae, and the way in which they have changed in response to zoo visitors.

Bovidae

10 papers investigated the impact of visitors on at least one bovid species with 14 different species/visitor variable/response to visitor combinations in total. In total there were 93 bovids included in the reviewed papers. There were ten species of bovid: domestic goats (*Capra hircus*), banteng (*Bos javanicus*), European bison (*Bison bonasus*), Soemmerring's Gazelle (*Gazella soemmerringii*), Indian blackbuck (*Antilope cervicapra*), Indian gaur (*Bos gaurus*), nyala (*Tragelaphus angasii*), Romanov sheep (*Ovis aries*) and waterbuck (*Kobus ellipsiprymnus*). The majority of responses to visitors were neutral (n=7) or negative (n=6). There was only one instance of a positive response and no responses were categorised as unknown. Number and presence/absence of visitors were the most frequently investigated measures (n=5 each). Visitor interactions were investigated in n=2 cases and visitor proximity and visitor noise were investigated in only one instance each. The animal-based indicators most frequently used across the bovids studied were: feeding (8% of observations), enclosure use (7%), negative social (9%), grooming (6%), positive social (13%), human-animal interactions (positive, negative and uncategorised) (16%) and resting (9%).

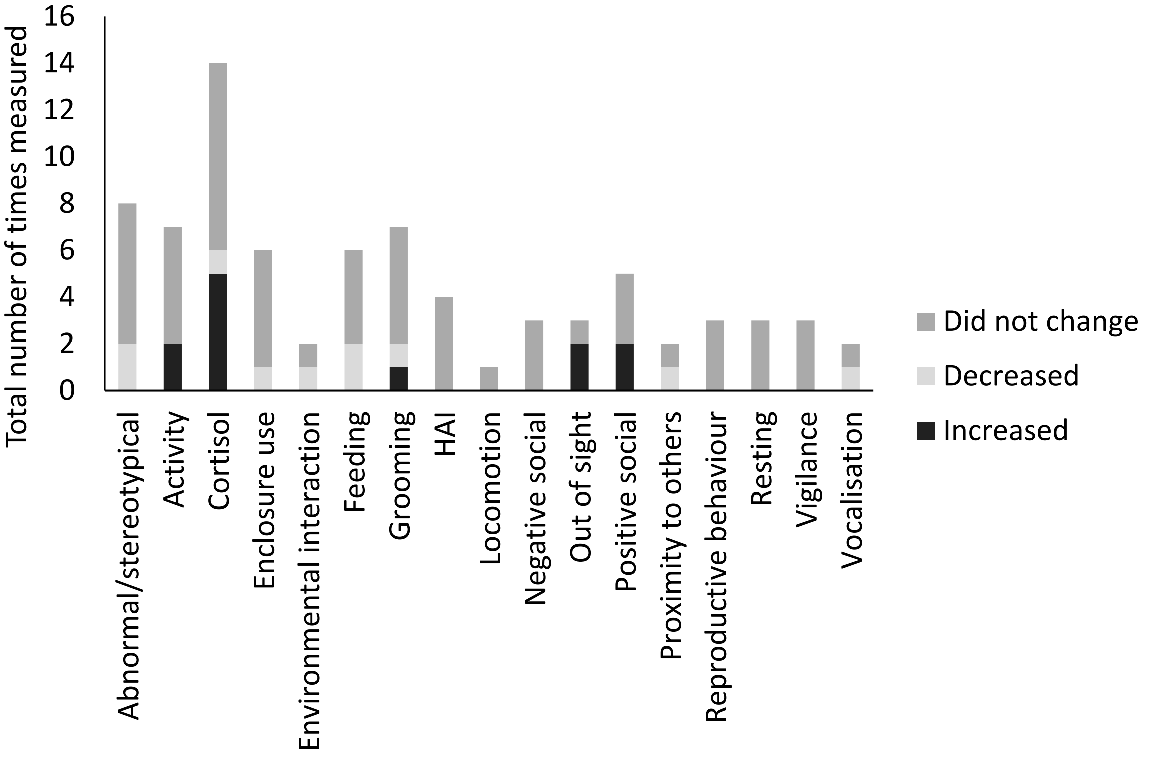

Figure S3. An overview of the animal-based indicators reported in the Bovidae, and the way in which they have changed in response to zoo visitors.

Macropodidae

Six papers investigated the impact of visitors on at least one macropodid species with 16 different species/visitor variable/response to visitor combinations in total. In total there were 93 macropodids included in the reviewed papers, which included five species: red kangaroo, western grey kangaroo (*Macropus fuliginosus*), quokka (*Setonix brachyurus*), red necked wallaby (*Macropus rufogriseus*) and swamp wallaby (*Wallabia bicolor*). Response to zoo visitors was predominantly neutral (n=10). There were only four negative and two 'unknown' responses reported. Visitor number was the most

frequently reported metric (n=7). Visitor noise and presence/absence of visitors was investigated in n=4 instances and visitor proximity in just one. The animal-based indicators most frequently used across the macropodids studied were: feeding (15.5%), enclosure use (14.4%), grooming (8.2%), positive social (7.2%), vigilance (15.5%), locomotion (7.2%) and resting (13.4%).

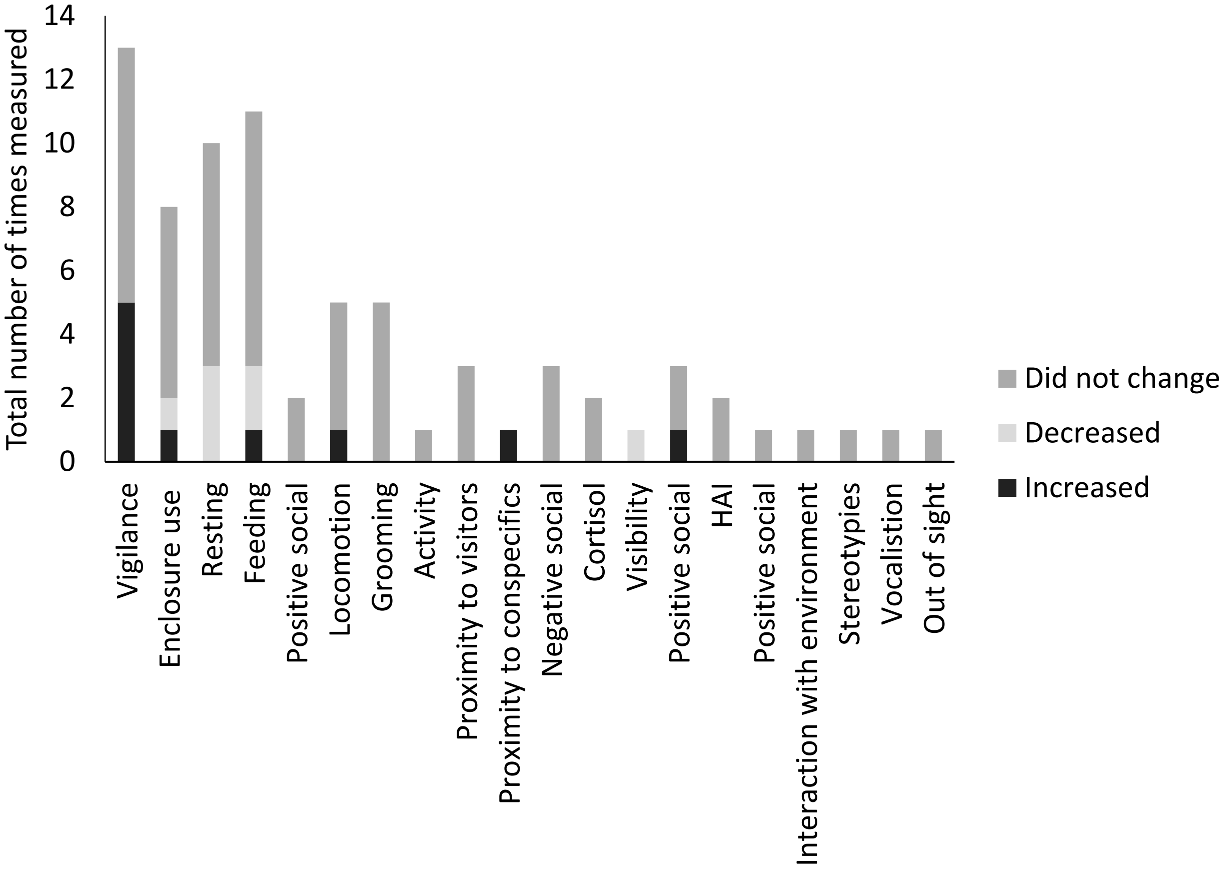

Figure S4. An overview of the animal-based indicators reported in the Macropodidae, and the way in which they have changed in response to zoo visitors.

Ursidae

Seven papers investigated the impact of visitors on at least one ursid species with 12 different species/visitor variable/response to visitor combinations in total. In total there were 33 ursids included in the reviewed papers which ranged seven species: brown bears (*Ursus arctos*), polar bears (*Ursus maritimus*), Andean bears (*Tremarctos ornatus*), sloth bears (*Melursus ursinus*), sun bears (*Helarctos malayanus*), black bears (*Ursus americanus*) and giant panda. The most frequent responses to visitors were neutral (n=4) and negative (n=4). Unknown responses were reported twice and positive once. One paper reported a neutral-positive response for one of the study bears. Visitor number and presence/absence of visitors were the most frequently reported visitor variables (n=5). Visitor noise was investigated in two instances. The animal-based indicators most frequently used across the ursids studied were: cortisol (6.1%), feeding (6.1%), enclosure use (10.2%), interaction with the environment (12.2%), positive social (6.1%), vigilance (6.1%), locomotion (8.2%), stereotypical/abnormal (16.3%) and activity/inactivity (10.2%).

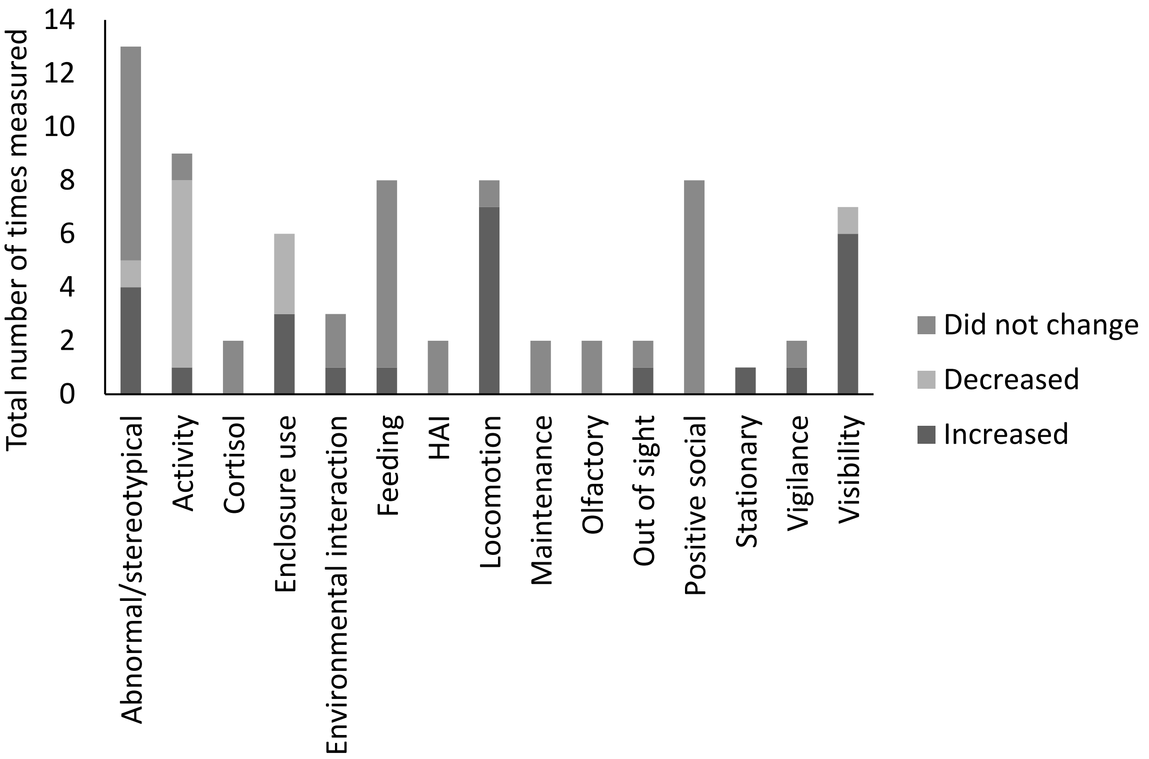

Figure S5. An overview of the animal-based indicators reported in the Ursidae, and the way in which they have changed in response to zoo visitors.

Birds

71 birds were included in the reviewed papers. These spanned 22 orders and encompassed 59 separate species from 32 families. The most frequently represented were African penguin (*Spheniscus demersus*) (n=5), little penguins (*Eudyptula minor*) (n=4) and Chilean flamingos (*Phoenicopterus chilensis*) (n=3). The most represented families (representing more than 5% of the birds studied) were: Anatidae (7%), Bucerotidae (7%), Phoenicopteridae (11.3%), Spheniscidae (18.3%), Threskiornithidae (5.6%). An overview of response to different visitor characteristics at a family level (for Anatidae, Bucerotidae, Phoenicopteridae, Spheniscidae and Threskiornithidae) is available online at https://tinyurl.com/yc68ppuw.

Anatidae

Two papers investigated the impact of visitors on at least Anatidae with 5 different species/visitor variable/response to visitor combinations in total. In total there were five different species of anatid: Madagascar teal (*Anas bernieri*), black-bellied whistling duck (*Dendrocygna autumnalis*), hottentot teal (*Spatula hottentota*), mandarin duck (*Aix galericulata*) and spotted whistling duck (*Dendrocygna guttata*). The most frequent response to visitors was neutral (n=4). An ‘unclear’ response was reported once. Visitor numbers were investigated once and visitor noise was investigated four times. The only animal-based measures used were enclosure use (50%), stereotypical/abnormal behaviour (40%) and activity/inactivity (10%).

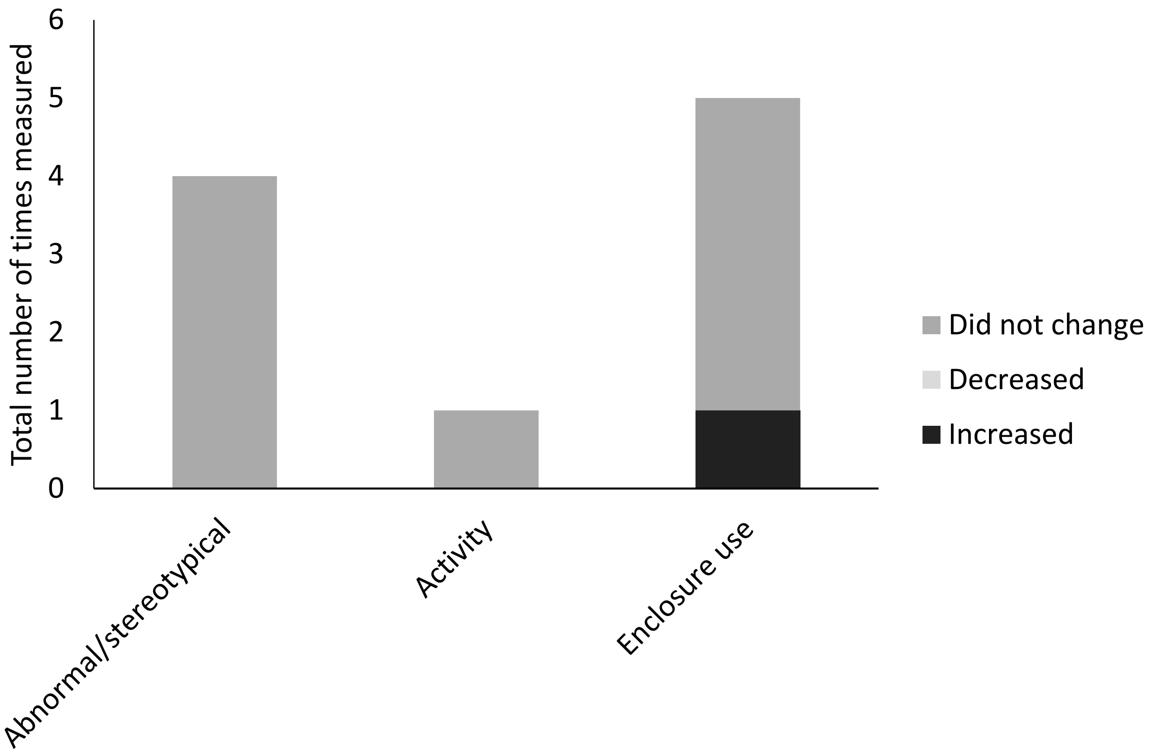

Figure S6. An overview of the animal-based indicators reported in the Anatidae, and the way in which they have changed in response to zoo visitors.

Bucerotidae

The impact of visitors on species within the Bucerotidae was assessed in four papers with 5 different species/visitor variable/response to visitor combinations in total. In total there were five different species: black casqued hornbill (*Ceratogymna atrata*), Indian hornbill (*Buceros bicornis*), Papuan wreathed hornbills (*Rhyticeros plicatus*), wrinkled hornbills (*Aceros corrugatus*) and Von der Decken's hornbills (*Tockus deckeni*). Responses to visitors were either neutral (n=3) or unclear (n=2). Visitor number was investigated three times, noise was investigated once and whether or not an event was on was also investigated only once. 12 different animal-based measures were used to assess the impact of visitors on members of the Bucerotidae. The most numerous were feeding and grooming (both 18.9%). Interaction with the environment accounted for 10.8% of the measures used across the studies. Other measures accounting for more than 5% were enclosure use (8.1%), activity/inactivity (8.1%), negative social (8.1%), positive social (5.4%), locomotion (5.4%), vocalisation (5.4%) and reproduction/maternal (5.4%).

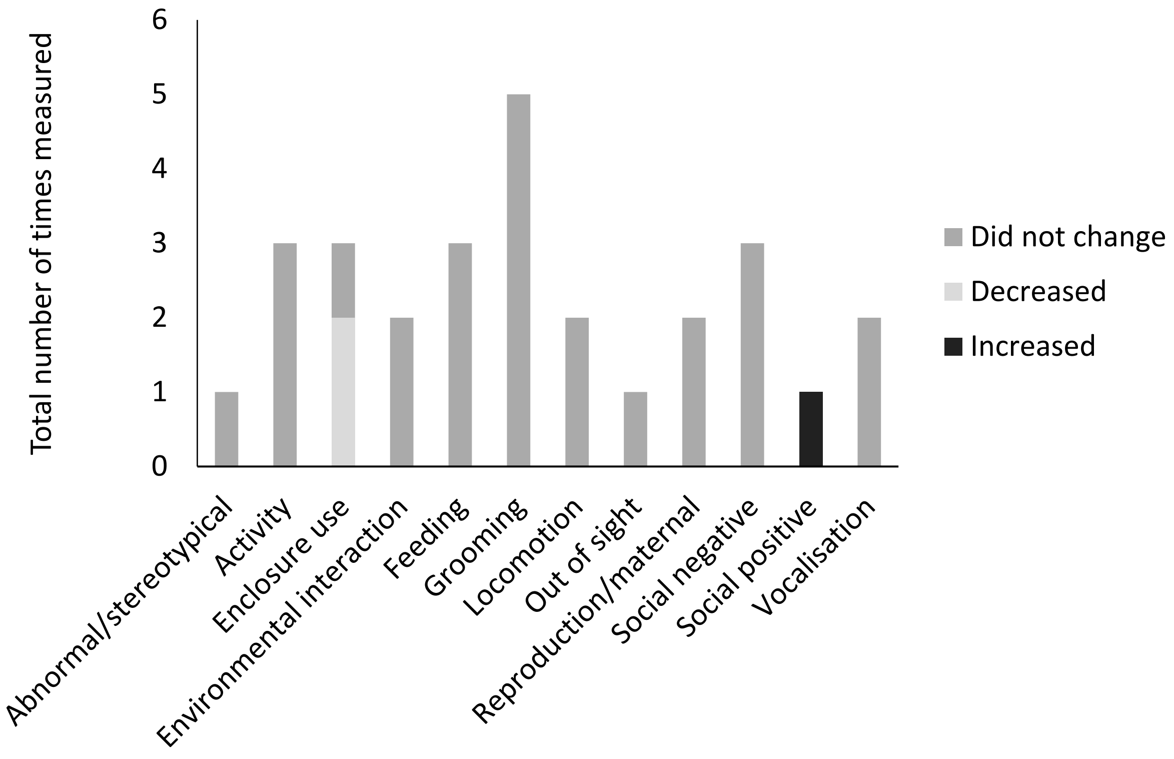

Figure S7. An overview of the animal-based indicators reported in the Bucerotidae, and the way in which they have changed in response to zoo visitors.

Phoenicopteridae

Members of the Phoenicopteridae were represented in three papers. There were five species: Chilean flamingo, greater flamingo (*Phoenicopterus roseus*), Andean flamingo (*Phoenicoparrus andinus*), lesser flamingo (*Phoeniconaias minor*) and Caribbean flamingos (*Phoenicopterus ruber*). There were nine different species/visitor variable/response to visitor combinations. The facility being open/closed was assessed in five instances and the number of visitors (number in the zoo or number at the enclosure) was assessed in four. Responses to visitors were predominantly neutral (n=7). In one instance a negative response was recorded and in one instance it was recorded as unknown. Only eight animal-based measures were used to assess the impact of visitors on phoenicopterids. All of these accounted for over 5% of the measures used. The most numerous were enclosure use (32.1%), activity/inactivity (17.9%) and proximity to conspecifics (14.3%). Grooming, feeding, locomotion, vigilance and resting were all assessed twice (7.1%).

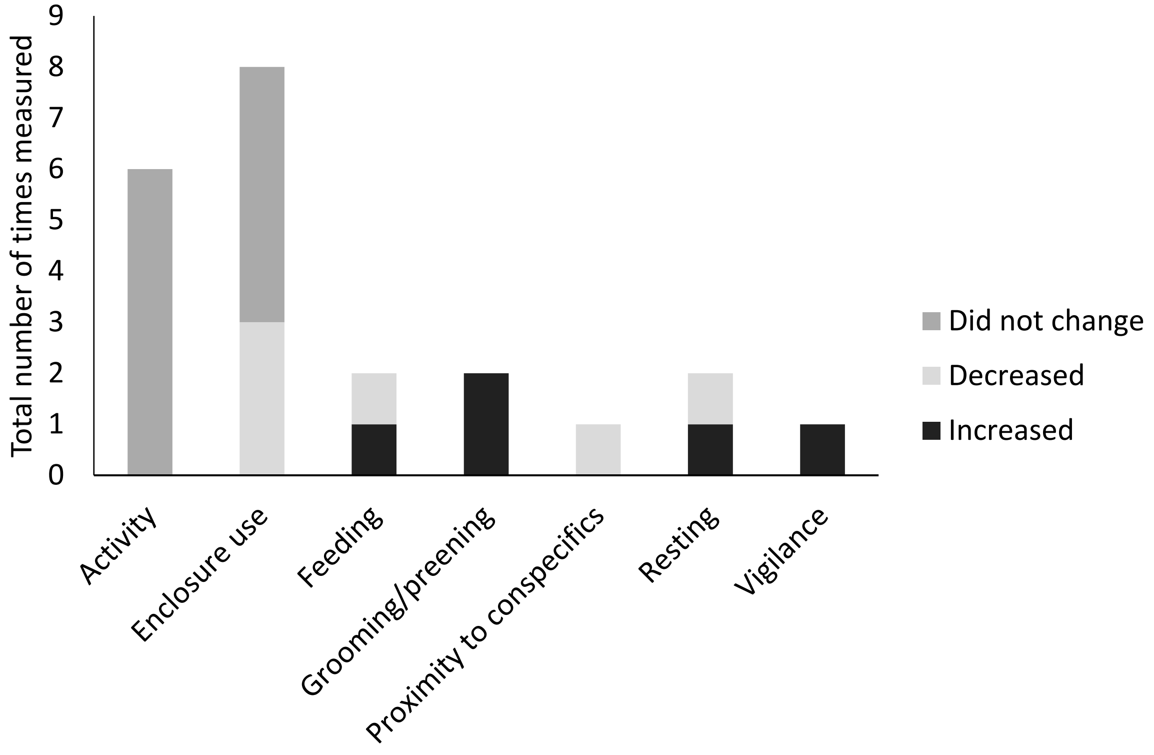

Figure S8. An overview of the animal-based indicators reported in the Phoenicopteridae, and the way in which they have changed in response to zoo visitors.

Spheniscidae

Spheniscidae were the most widely studied bird species. 11 papers investigated the impact of visitors on spheniscids with 13 different species/visitor variable/response to visitor combinations in total. In total there were six species of penguin: little penguins, African penguins, Humboldt penguins (*Spheniscus humboldti*), gentoo penguins (*Pygoscelis papua*), magellanic penguins (*Spheniscus magellanicus*) and Fiordland penguins (*Eudyptes pachyrhynchus*). The most frequent response to visitors was negative (n=4) and neutral (n=3). A report of the valence of the impact of visitors being unknown was found in two assessments and one study found there to be a positive impact of visitors. There were a further two studies who proclaimed visitors had a neutral-negative impact from visitors and one which identified a neutral/positive impact on zoo animals. Number and presence/absence of visitors were the most frequently investigated metrics, with n=6 and n=3 investigations respectively. Visitor proximity, visitor encounters and events in the zoo were also investigated but these were reported more infrequently; once, twice and once respectively. There were 21 separate animal-based measures applied to studies investigating the impact of visitors on the Spheniscidae family. The most frequently used were grooming (10.3%), positive social (11.2%) and swimming (11.2%). As a water-based family, swimming is likely also representative of animal locomotion and enclosure use, so monitoring this behaviour captures other metrics too. Other animal-based measures which were accounted for over 5% of the animal-based measures used across the studies were enclosure use (5.2%), interaction with the environment (5.2%), resting (5.2%), locomotion (6%), vigilance (6%), HAIs (8.6%) and negative social (8.6%).

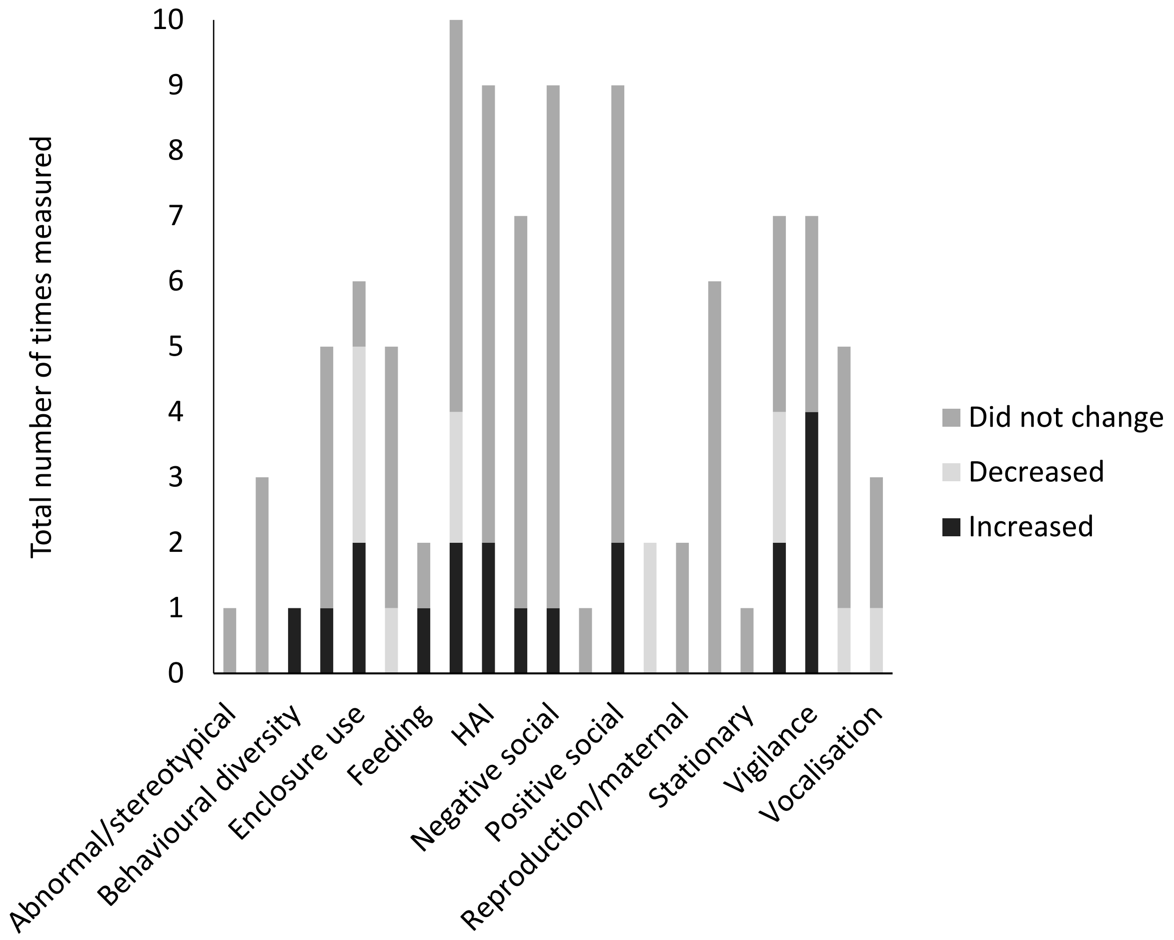

Figure S9. An overview of the animal-based indicators reported in the Spheniscidae, and the way in which they have changed in response to zoo visitors.

Threskiornithidae

The impact of visitors on species within the Threskiornithidae was assessed in just one paper with eight different species/visitor variable/response to visitor combinations in total. In total there were three different species: scarlet ibis (*Eudocimus ruber*), sacred ibis (*Threskiornis aethiopicus*) and African spoonbills (*Platalea alba*). Responses to visitors were all categorised as neutral (n=8). Visitor number and visitor noise were both investigated four times within the study. The only two animal-based measures used were stereotypical/abnormal behavior and enclosure use. These were used equally and therefore accounted for 50% of the recorded animal-based measures each. In the six times these measures were used, they did not change in relation to zoo visitors.

Fish

Ten fish were included in the reviewed papers. These spanned 6 orders and encompassed 9 separate species from 8 families. The most frequently represented were cownose ray (*Rhinoptera bonasus*) (n=2). All other species were represented only once: banded morwong (*Cheilodactylus spectabilis*), bonnethead shark (*Sphyrna tiburo*), brownbanded bamboo shark (*Chiloscyllium punctatum*), moonlighter (*Tilodon sexfasciatus*), Port Jackson shark (*Heterodontus portusjacksoni*), southern fiddler

ray (*Trygonorrhina dumerilii*), southern stingray (*Hypanus* americanus) and white-spotted bamboo shark (*Chiloscyllium plagiosum*). The most represented families were: Hemiscyllidae (20%) and Rhinopteridae (20%). All other families, Cheilodactylidae, Dasyatidae, Heterodontidae, Kyphosidae, Rhinobatidae and Sphyrnidae each represented 10% of fish species studied. An overview of response to different visitor characteristics at a family level for the two most represented families is available online at https://tinyurl.com/yc68ppuw.

Hemiscyllidae

The impact of visitors on species within the Hemiscyllidae was assessed in just one paper, with two species investigated: brownbanded bamboo shark and white-spotted bamboo shark. There were two different species/visitor variable/response to visitor combinations. Both species were investigated only in relation to a visitor interaction programme. Responses to visitors were classified as unclear for both species. The four animal-based measures used were swimming, interaction with the environment, resting and enclosure use. These were used equally and thus accounted for 25% each of the measures. Enclosure use (n=3), environmental interaction (n=1), resting (n=3) and swimming (n=1) were recorded. No changes were observed in any of the behaviours.

Rhinopteridae

The impact of visitors on species within the Rhinopteridae was assessed in just one paper with just one species investigated, cownose ray. There were two different species/visitor variable/response to visitor combinations, with the rays being investigated in relation to a visitor interaction programme and number of visitors at the enclosure. As with the Hemiscyllidae*, r*esponse to visitors was classed as unknown. Six animal-based measures were used: solitary behaviour, social behaviour, swimming, interaction with enrichment, resting and enclosure use. Each one was assessed once per visitor condition for the study species.

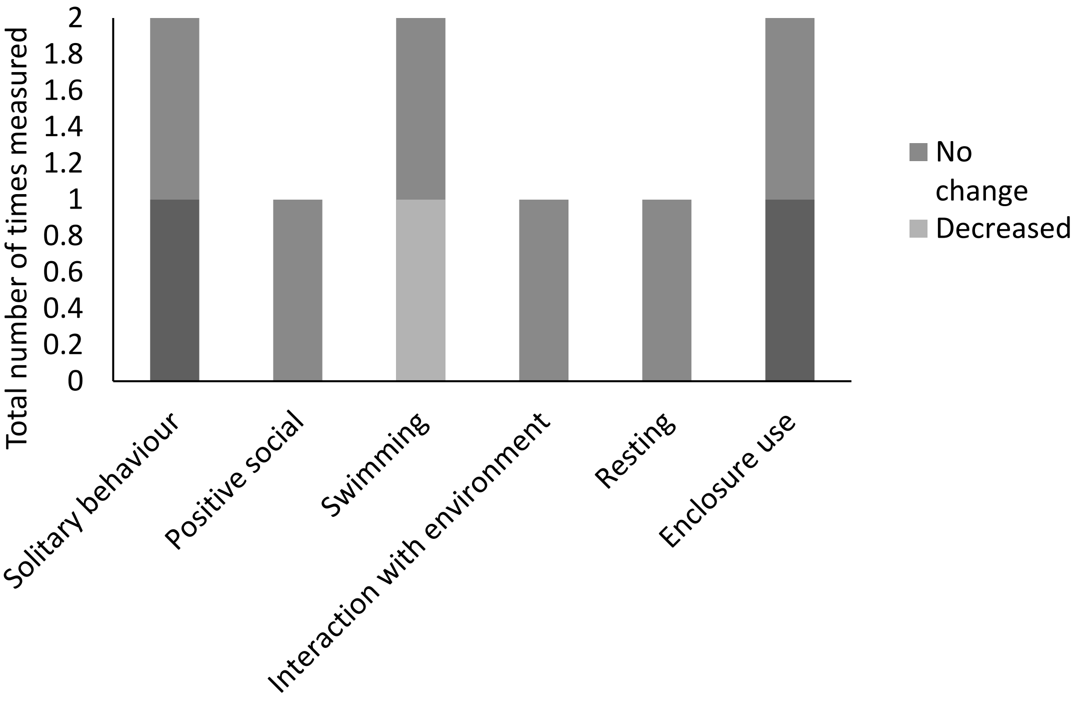

Figure S10. An overview of the animal-based indicators reported in the Rhinopteridae, and the way in which they have changed in response to zoo visitors.

Amphibians

Five amphibians were included in the reviewed papers. These spanned one order and encompassed five separate species from four families. All amphibian species were represented only once: common frog (*Rana temporaria*), common toad (*Bufo bufo*), golden mantilla (*Mantella aurantiaca*), golden poison dart frog (*Phyllobates terribilis*) and a pool frog (*Pelophylax lessonae*). The families represented were Bufonidae (20%), Dendrobatidae (20%), Mantellidae (20%) and Ranidae (40%). An overview of response to different visitor characteristics at a family level for these families is available online at https://tinyurl.com/yc68ppuw.

All of the amphibians included in this review were contained within the same paper. There was one species represented in the Bufonidae, Dendrobatidae and Mantellidae: common toad, golden poison dart frog and golden Mantella respectively. Ranidae had two species; the common frog and the pool frog. All of these species were studied in relation to the presence/absence of visitors, and visibility was the only animal-based measure used. In all instances the visibility of the animals decreased. For all species the impact of visitors was identified as negative, however in the Bufonidae this was highlighted by the authors as being a temporary response to the return of visitors after the COVID-19 pandemic.

Reptiles

22 reptiles were included in the reviewed papers. These spanned 4 orders and encompassed 22 separate species from 18 families. Each reptile species was represented only once. The most represented families (representing more than 5% of the reptiles studied) were: Elapidae (9.1%), Helodermatidae (9.1%), Pythonidae (9.1%) and Scincidae (9.1%). An overview of response to different visitor characteristics at a family level for these families is available online at https://tinyurl.com/yc68ppuw.

Elapidae

There were two species of elapid which were recorded within one paper: king cobra (*Ophiophagus hannah*) and inland taipan (*Oxyuranus microlepidotus*). The visitor variable assessed was visitor presence/absence. Only activity and whether or not the animal was in sight were recorded for elapid species. Neither of these measures changed in the twice they were each used and the impact of visitors was identified as neutral in both cases.

Helodermatidae

There were two species of helodermatid (beaded lizards (*Heloderma horridum*) and Rio Fuerte beaded lizards (*Heloderma exasperatum*)) which were assessed in two papers. Out of sight, behavioural diversity, enclosure use, environmental interaction, positive social interactions and visibility were all recorded once. Activity was recorded twice. Out of sight decreased and positive social interactions increased in relation to zoo visitors. All other metrics did not change in relation to zoo visitors. The impact of visitors on beaded lizards was classed as unclear whilst the response to the Rio Fuerte beaded lizard was identified as neutral.

Pythonidae

Pythonid species were assessed in one paper. There were two species studied from the Pythonidae: reticulated python (*Malayopython reticulatus*) and jungle python (*Morelia spilota*). The visitor variable assessed in relation to these species was the presence/absence of visitors. Only activity and whether

or not the animal was in sight were recorded. Neither of these measures changed in the twice they were each used. In both instances the impact of visitors was considered to be neutral.

Scincidae

There were two scinid species assessed: gidgee skink (*Egernia stokesii*) and Telfair's skink (*Leiolopisma telfairii*). One paper assessed the impact of the presence/absence of visitors on these two species. Only activity and whether or not the animal was in sight were recorded. Neither of these measures changed in the twice they were used. The impact of visitors on Scinidae was categorised as neutral.

Invertebrates

Invertebrate research is rather more limited, with only two papers being published. Three invertebrates were included in the reviewed papers. They spanned two orders and encompassed two separate species from two families in two different classes. Fiddler crabs were represented twice and the Mexican red-kneed spider (*Brachypelma hamorii*) once. The families represented were Ocypodidae (66.7%) and Theraphosidae (33.3%). An overview of response to different visitor characteristics at a family level for these families is available online at https://tinyurl.com/yc68ppuw.

Ocypodidae

The response of two fiddler crab species, the mudflat fiddler crab (*Uca rapax*) and the west African fiddler crab (*Uca tangeri*) to the number of visitors was assessed in one paper. Only one indicator was measured in the Ocypodidae, visibility. This did not change in relation to visitors and the impact of visitors on the two species was identified as neutral.

Theraphosidae

Only one species of spider was assessed within the Theraphosidae, the Mexican red-kneed spider. This was analysed within one paper. The visitor variable of interest was response to handling by visitors. Four indicators were used to assess the theraphosids: grooming, locomotion, environmental interaction and enclosure use. Enclosure use and grooming increased. Locomotion and environmental interaction did not change. The impact of visitors on this species was identified as being unclear.
