# Supplementary material for: The Impact of Visitors on Non-Primate Species in Zoos: A Quantitative Review
Source: Animals (Basel). 2023 Mar 28;13(7):1178. doi: 10.3390/ani13071178 (PMC10092984; doi:10.3390/ani13071178)
Supplement: Supplementary file 1 [file animals-13-01178-s001.zip › Williams et al_supplementary material_S2.pdf]

Supplementary material

File 2: Details on the way in which each animal-based measure changed in the different taxa or animal categories.

Table S1. Details on the way in which each animal-based measures changed in amphibians

**Behaviour**	**Amphibians**						
	**Changed**	**Closer**	**Further**	**Decreased**	**Increased**	**None**	**Percentage changed**
Abrupt behaviour	0	0	0	0	0	0	0.0
Activity/ inactivity	0	0	0	0	0	0	0.0
Behavioural diversity	0	0	0	0	0	0	0.0
Breathing rate	0	0	0	0	0	0	0.0
Bodyweight	0	0	0	0	0	0	0.0
Cortisol/ corticosteroids	0	0	0	0	0	0	0.0
Enclosure use	0	0	0	0	0	0	0.0
Feeding	0	0	0	0	0	0	0.0
Grooming	0	0	0	0	0	0	0.0
HAI	0	0	0	0	0	0	0.0
HAI negative	0	0	0	0	0	0	0.0
HAI positive	0	0	0	0	0	0	0.0
Heart rate	0	0	0	0	0	0	0.0
Interaction with environment	0	0	0	0	0	0	0.0
Locomotion	0	0	0	0	0	0	0.0
Maintenance	0	0	0	0	0	0	0.0
Negative social	0	0	0	0	0	0	0.0
Nervous	0	0	0	0	0	0	0.0
Olfactory	0	0	0	0	0	0	0.0
OOS	0	0	0	0	0	0	0.0
Other	0	0	0	0	0	0	0.0
Positive social	0	0	0	0	0	0	0.0
Proximity to conspecifics	0	0	0	0	0	0	0.0
Proximity to visitors	0	0	0	0	0	0	0.0
Reactivity to people	0	0	0	0	0	0	0.0
Reproduction/ maternal	0	0	0	0	0	0	0.0

Resting	0	0	0	0	0	0	0.0
Solitary behaviour	0	0	0	0	0	0	0.0
Species typical	0	0	0	0	0	0	0.0
Stationary	0	0	0	0	0	0	0.0
Stereotypical/ abnormal	0	0	0	0	0	0	0.0
Swimming	0	0	0	0	0	0	0.0
Vigilance	0	0	0	0	0	0	0.0
Visibility	0	0	0	5	0	0	100.0
Vocalisation	0	0	0	0	0	0	0.0

Table S2. Details on the way in which each animal-based measures changed in birds

**Behaviour**	**Birds**						
	**Changed**	**Closer**	**Further**	**Decreased**	**Increased**	**None**	**Percentage changed**
Abrupt behaviour	0	0	0	0	0	0	0.0
Activity/ inactivity	0	0	0	1	5	27	18.2
Behavioural diversity	0	0	0	0	1	0	100.0
Breathing rate	0	0	0	0	0	0	0.0
Bodyweight	0	0	0	0	0	0	0.0
Cortisol/ corticosteroids	0	0	0	0	2	4	33.3
Enclosure use	8	3	7	3	2	60	27.7
Feeding	0	0	0	1	2	22	12.0
Grooming	0	0	0	3	2	30	14.3
HAI	0	0	0	0	0	3	0.0
HAI negative	0	0	0	0	1	2	33.3
HAI positive	0	0	0	1	1	4	33.3
Heart rate	0	0	0	0	0	0	0.0
Interaction with environment	0	0	0	1	0	17	5.6
Locomotion	0	0	0	1	2	20	13.0
Maintenance	0	0	0	1	0	0	100.0
Negative social	0	0	0	0	2	18	10.0
Nervous	0	0	0	0	0	0	0.0
Olfactory	0	0	0	0	0	0	0.0
OOS	0	0	0	0	0	9	0.0
Other	0	0	0	1	1	55	3.5
Positive social	0	0	0	0	5	16	23.8

Proximity to conspecifics	0	0	0	2	1	1	75.0
Proximity to visitors	0	0	0	0	2	0	100.0
Reactivity to people	0	0	0	0	0	0	0.0
Reproduction/ maternal	0	0	0	0	0	10	0.0
Resting	0	0	0	8	3	16	40.7
Solitary behaviour	0	0	0	0	0	0	0.0
Species typical	0	0	0	0	0	2	0.0
Stationary	0	0	0	0	0	2	0.0
Stereotypical/ abnormal	0	0	0	1	2	61	4.7
Swimming	0	0	0	2	3	8	38.5
Vigilance	0	0	0	0	7	8	46.7
Visibility	0	0	0	1	0	7	12.5
Vocalisation	0	0	0	1	1	10	16.7

Table S3. Details on the way in which each animal-based measures changed in fish

**Behaviour**	**Fish**						
	**Changed**	**Closer**	**Further**	**Decreased**	**Increased**	**None**	**Percentage changed**
Abrupt behaviour	0	0	0	0	0	0	0.0
Activity/ inactivity	0	0	0	0	0	1	0.0
Behavioural diversity	0	0	0	0	0	0	0.0
Breathing rate	0	0	0	0	0	0	0.0
Bodyweight	0	0	0	0	0	0	0.0
Cortisol/ corticosteroids	0	0	0	0	0	0	0.0
Enclosure use	1	0	3	0	2	8	42.9
Feeding	0	0	0	0	0	0	0.0
Grooming	0	0	0	0	0	0	0.0
HAI	0	0	0	0	0	0	0.0
HAI negative	0	0	0	0	0	0	0.0
HAI positive	0	0	0	0	0	0	0.0
Heart rate	0	0	0	0	0	0	0.0
Interaction with environment	0	0	0	0	0	6	0.0
Locomotion	0	0	0	0	0	0	0.0

Maintenance	0	0	0	0	0	0	0.0
Negative social	0	0	0	0	0	0	0.0
Nervous	0	0	0	0	0	0	0.0
Olfactory	0	0	0	0	0	0	0.0
OOS	0	0	0	0	0	0	0.0
Other	0	0	0	0	0	0	0.0
Positive social	0	0	0	0	0	2	0.0
Proximity to conspecifics	0	0	0	0	0	0	0.0
Proximity to visitors	0	0	0	0	0	0	0.0
Reactivity to people	0	0	0	0	0	0	0.0
Reproduction/ maternal	0	0	0	0	0	0	0.0
Resting	0	0	0	2	1	7	30.0
Solitary behaviour	0	0	0	0	1	1	50.0
Species typical	0	0	0	0	0	0	0.0
Stationary	0	0	0	0	0	0	0.0
Stereotypical/ abnormal	0	0	0	0	0	4	0.0
Swimming	0	0	0	2	3	8	38.5
Vigilance	0	0	0	0	0	0	0.0
Visibility	0	0	0	0	0	0	0.0
Vocalisation	0	0	0	0	0	0	0.0

Table S4. Details on the way in which each animal-based measures changed in invertebrates

**Behaviour**	**Invertebrates**						
	**Changed**	**Closer**	**Further**	**Decreased**	**Increased**	**None**	**Percentage changed**
Abrupt behaviour	0	0	0	0	0	0	0.0
Activity/ inactivity	0	0	0	0	0	0	0.0
Behavioural diversity	0	0	0	0	0	0	0.0
Breathing rate	0	0	0	0	0	0	0.0
Bodyweight	0	0	0	0	0	0	0.0
Cortisol/ corticosteroids	0	0	0	0	0	0	0.0
Enclosure use	1	0	0	0	0	0	100.0
Feeding	0	0	0	0	0	0	0.0
Grooming	0	0	0	0	1	0	100.0
HAI	0	0	0	0	0	0	0.0
HAI negative	0	0	0	0	0	0	0.0

HAI positive	0	0	0	0	0	0	0.0
Heart rate	0	0	0	0	0	0	0.0
Interaction with environment	0	0	0	0	0	1	0.0
Locomotion	0	0	0	0	0	1	0.0
Maintenance	0	0	0	0	0	0	0.0
Negative social	0	0	0	0	0	0	0.0
Nervous	0	0	0	0	0	0	0.0
Olfactory	0	0	0	0	0	0	0.0
OOS	0	0	0	0	0	0	0.0
Other	0	0	0	0	0	0	0.0
Positive social	0	0	0	0	0	0	0.0
Proximity to conspecifics	0	0	0	0	0	0	0.0
Proximity to visitors	0	0	0	0	0	0	0.0
Reactivity to people	0	0	0	0	0	0	0.0
Reproduction/ maternal	0	0	0	0	0	0	0.0
Resting	0	0	0	0	0	0	0.0
Solitary behaviour	0	0	0	0	0	0	0.0
Species typical	0	0	0	0	0	0	0.0
Stationary	0	0	0	0	0	0	0.0
Stereotypical/ abnormal	0	0	0	0	0	0	0.0
Swimming	0	0	0	0	0	0	0.0
Vigilance	0	0	0	0	0	0	0.0
Visibility	0	0	0	0	0	2	0.0
Vocalisation	0	0	0	0	0	0	0.0

Table S5. Details on the way in which each animal-based measures changed in mammals

**Behaviour**	**Mammals**						
	**Changed**	**Closer**	**Further**	**Decreased**	**Increased**	**None**	**Percentage changed**
Abrupt behaviour	0	0	0	1	15	0	100.0
Activity/ inactivity	0	0	0	22	0	42	34.4
Behavioural diversity	0	0	0	1	0	0	100.0
Breathing rate	0	0	0	0	1	1	50.0
Bodyweight	0	0	0	1	0	0	100.0

Cortisol/ corticosteroids	0	0	0	1	14	12	55.6
Enclosure use	2	7	7	2	2	37	35.1
Feeding	0	0	0	9	9	61	22.8
Grooming	0	0	0	2	4	46	11.5
HAI	0	0	0	1	2	5	37.5
HAI negative	0	0	0	0	5	31	13.9
HAI positive	0	0	0	2	5	23	23.3
Heart rate	0	0	0	1	0	1	50.0
Interaction with environment	0	0	0	6	4	33	23.3
Locomotion	0	0	0	6	9	61	19.7
Maintenance	0	0	0	0	0	2	0.0
Negative social	0	0	0	3	4	52	11.9
Nervous	0	0	0	0	0	2	0.0
Olfactory	0	0	0	0	1	15	6.3
OOS	0	0	0	1	3	49	7.5
Other	0	0	0	0	1	21	4.5
Positive social	0	0	0	7	5	71	14.5
Proximity to conspecifics	0	0	0	1	2	7	30.0
Proximity to visitors	0	0	0	5	1	4	60.0
Reactivity to people	0	0	0	0	1	0	100.0
Reproduction/ maternal	0	0	0	1	0	13	7.1
Resting	0	0	0	19	10	119	19.6
Solitary behaviour	0	0	0	0	0	0	0.0
Species typical	0	0	0	0	0	0	0.0
Stationary	0	0	0	1	2	4	42.9
Stereotypical/ abnormal	0	0	0	11	14	71	26.0
Swimming	0	0	0	1	3	3	57.1
Vigilance	0	0	0	6	16	54	28.9
Visibility	0	0	0	5	3	5	61.5
Vocalisation	0	0	0	0	1	18	5.3

Table S6. Details on the way in which each animal-based measures changed in reptiles

**Behaviour**	**Reptiles**						
	**Changed**	**Closer**	**Further**	**Decreased**	**Increased**	**None**	**Percentage changed**
Abrupt behaviour	0	0	0	0	0	0	0.0

Activity/ inactivity	0	0	0	4	4	14	36.4
Behavioural diversity	0	0	0	1	2	3	50.0
Breathing rate	0	0	0	0	0	0	0.0
Bodyweight	0	0	0	0	0	0	0.0
Cortisol/ corticosteroids	0	0	0	0	0	0	0.0
Enclosure use	0	0	0	1	0	10	9.1
Feeding	0	0	0	0	0	3	0.0
Grooming	0	0	0	0	0	0	0.0
HAI	0	0	0	0	0	0	0.0
HAI negative	0	0	0	0	0	0	0.0
HAI positive	0	0	0	0	0	0	0.0
Heart rate	0	0	0	0	0	0	0.0
Interaction with environment	0	0	0	0	1	6	14.3
Locomotion	0	0	0	0	0	3	0.0
Maintenance	0	0	0	0	0	1	0.0
Negative social	0	0	0	1	1	0	100.0
Nervous	0	0	0	0	0	0	0.0
Olfactory	0	0	0	0	0	0	0.0
OOS	0	0	0	0	1	10	9.1
Other	0	0	0	0	0	2	0.0
Positive social	0	0	0	1	2	5	37.5
Proximity to conspecifics	0	0	0	1	0	0	100.0
Proximity to visitors	0	0	0	0	0	0	0.0
Reactivity to people	0	0	0	0	0	0	0.0
Reproduction/ maternal	0	0	0	0	0	0	0.0
Resting	0	0	0	0	0	2	0.0
Solitary behaviour	0	0	0	0	0	0	0.0
Species typical	0	0	0	0	0	0	0.0
Stationary	0	0	0	0	0	0	0.0
Stereotypical/ abnormal	0	0	0	0	0	0	0.0
Swimming	0	0	0	0	0	0	0.0
Vigilance	0	0	0	0	0	1	0.0
Visibility	0	0	0	5	1	10	37.5
Vocalisation	0	0	0	0	0	1	0.0
